# An overview of the two-component system GarR/GarS role on antibiotic production in *Streptomyces coelicolor*

**DOI:** 10.1007/s00253-024-13136-z

**Published:** 2024-04-24

**Authors:** Rodrigo Cruz-Bautista, Augusto Zelarayan-Agüero, Beatriz Ruiz-Villafán, Adelfo Escalante-Lozada, Romina Rodríguez-Sanoja, Sergio Sánchez

**Affiliations:** 1 Departamento de Biología Molecular y Biotecnología, Instituto de Investigaciones Biomédicas, Universidad Nacional Autónoma de México, Ciudad Universitaria, 04510 Mexico City, Mexico; 2 Departamento de Ingeniería Celular y Biocatálisis, Instituto de Biotecnología, Universidad Nacional Autónoma de México, Ave. Universidad 2001, 62210 Cuernavaca, Mexico

**Keywords:** *Streptomyces*, Two-component systems, Antibiotics, Regulation, Sensor histidine kinase, Response regulator

## Abstract

**Abstract:**

The *Streptomyces* genus comprises Gram-positive bacteria known to produce over two-thirds of the antibiotics used in medical practice. The biosynthesis of these secondary metabolites is highly regulated and influenced by a range of nutrients present in the growth medium. In *Streptomyces coelicolor*, glucose inhibits the production of actinorhodin (ACT) and undecylprodigiosin (RED) by a process known as carbon catabolite repression (CCR). However, the mechanism mediated by this carbon source still needs to be understood. It has been observed that glucose alters the transcriptomic profile of this actinobacteria, modifying different transcriptional regulators, including some of the one- and two-component systems (TCSs). Under glucose repression, the expression of one of these TCSs SCO6162/SCO6163 was negatively affected. We aimed to study the role of this TCS on secondary metabolite formation to define its influence in this general regulatory process and likely establish its relationship with other transcriptional regulators affecting antibiotic biosynthesis in the *Streptomyces* genus. In this work, in silico predictions suggested that this TCS can regulate the production of the secondary metabolites ACT and RED by transcriptional regulation and protein–protein interactions of the transcriptional factors (TFs) with other TCSs. These predictions were supported by experimental procedures such as deletion and complementation of the TFs and qPCR experiments. Our results suggest that in the presence of glucose, the TCS SCO6162/SCO6163, named GarR/GarS, is an important negative regulator of the ACT and RED production in *S. coelicolor*.

**Key points:**

*• GarR/GarS is a TCS with domains for signal transduction and response regulation*

*• GarR/GarS is an essential negative regulator of the ACT and RED production*

*• GarR/GarS putatively interacts with and regulates activators of ACT and RED*

**Supplementary Information:**

The online version contains supplementary material available at 10.1007/s00253-024-13136-z.

## Introduction

The Actinobacteria phylum includes the *Streptomyces* genus (Oren and Garrity 2021), a Gram-positive bacteria with a linear chromosome ranging from 6.7 to 12.3 Mb and a high GC DNA content (Chater 2016). These microorganisms can be found in various terrestrial and aquatic environments, including extreme habitats, and are known to produce many secondary metabolites, including over two-thirds of the antibiotics used in medical practice (Donald et al. 2022). Under laboratory conditions, *Streptomyces coelicolor* has been adopted as one of the models for regulatory studies on antibiotic biosynthesis because of its amenability to genetic analysis and rapid response detection due to its ability to produce two pigmented secondary metabolites known as actinorhodin (ACT) (Wright and Hopwood 1976) and undecylprodigiosin (RED) (Rudd and Hopwood 1980). The production of these and other secondary metabolites is influenced by environmental nutrients like carbon, nitrogen, and phosphate when available in the culture media (Romero-Rodríguez et al. 2016a; Krysenko 2023; Barreiro and Martínez-Castro 2019). The effect of the carbon source on the synthesis of secondary metabolites has been extensively studied in *Streptomyces* (Romero-Rodríguez et al. 2016a; Guzmán et al. 2005; Gubbens et al. 2012; Magdalena et al. 2012; Tierrafría et al. 2016), which is regulated by a mechanism known as carbon catabolite repression (CCR) (Ruiz-Villafán et al. 2021). Although glucose and the glucose kinase (Glk) are the most studied factors influencing this regulatory process in *S. coelicolor*, nowadays, its precise mechanism is still poorly understood (Ruiz-Villafán et al. 2021; Angell et al. 1994; Rocha-Mendoza et al. 2021). Glucose, a carbon source commonly used under laboratory conditions, promotes the rapid growth of *Streptomyces* and, at the same time, represses the synthesis of secondary metabolites (Romero-Rodríguez et al. 2018; Guzmán et al. 2005; Tierrafría et al. 2016). Environmental stimuli highly regulate these compounds through transcriptional factors (TFs) and protein systems in prokaryotes and lower eukaryotes, allowing cells to sense and respond to chemical or physical signals (Buschiazzo and Trajtenberg 2019). The two-component systems (TCSs) represent the most relevant signal transduction mechanism operating in *Streptomyces* (Cruz-Bautista et al. 2023). They comprise a sensor histidine kinase (SHK) and a cognate response regulator (RR). The regulatory mechanism begins when the SHK senses an environmental stimulus and transmits the signal by transferring a phosphate group from a conserved histidine to a conserved aspartate in the RR, which commonly binds to DNA and triggers a physiological response (Jacob-Dubuisson et al. 2018). Among bacteria, the *Streptomyces* genus holds the highest number of TCSs, reflecting their need to adapt rapidly to highly variable environmental changes and survive within their ecological niche (Jacob-Dubuisson et al. 2018). To date, 34 predicted TCSs in *S. coelicolor* have been studied and associated with different processes, including physiology, secondary metabolism, morphological differentiation, and some cellular processes (Jin et al. 2023). The interest in understanding the regulatory pathways in *Streptomyces* lies firstly in unveiling the mechanisms for antibiotic biosynthesis in this genus. Secondly, to understand the likely participation of various silent cryptic biosynthetic gene clusters (BGCs) in the regulation of secondary metabolite biosynthesis and their potential as targets for overproduction purposes (Cruz-Bautista et al. 2023; Sánchez de la Nieta et al. 2022).

The combination of bioinformatic tools and different experimental techniques has become a valuable tool in the study and understanding of the TCSs, allowing the generation of the necessary data to elucidate the role of these proteins in the regulatory mechanisms (Cruz-Bautista et al. 2023; van der Heul et al. 2018).

In this work, we focused on the *S. coelicolor* TCS SCO6162/SCO6163, which was observed to be downregulated under glucose-repressive conditions (Romero-Rodríguez et al. 2016b) and was renamed here as GarR/GarS (Glucose-responsive antibiotic regulator) due to its relationship with antibiotic production in response to glucose. By using bioinformatic methods complemented with experimental analysis on deleted *S. coelicolor* TCS mutants, we found that GarR/GarS putatively interact with and regulate activators of ACT (*actII-ORF4*) and RED (*redD* and *redZ*) at the transcriptional level. These estimations were supported by experimental procedures such as the deletion and complementation of the GarR/GarS and were tested by means of qPCR experiments. Our results suggest that GarR/GarS is an essential negative regulator of the ACT and RED production in *S. coelicolor* in the presence of glucose.

## Materials and methods

### Strains, plasmids, primers, and growth conditions

*Streptomyces coelicolor* M145 was used as a wild-type (WT) strain to generate TCS mutants. The WT strain and its derived mutants are available at the UNAM-48/WFCC (Mexico City). All *Streptomyces* strains were stored as spore suspensions in 20% (v/v) glycerol at − 20 °C as previously described (Rocha-Mendoza et al. 2021). Gene disruption was performed by PCR-targeting with the cosmid St1A9, *E*. *coli* BW25113 containing the vector pIJ790, and *E*. *coli* ET12567 containing the vector pUZ8002 (Gust et al. 2003). The pGEM®-T Easy Vector (Promega) was used for *garR* cloning, and the pKU1021 integrative vector was used as the backbone for mutant strain complementation. Minimal liquid medium (NMMP) (NH_4_)_2_SO_4_ 0.2%, Casamino acids 0.5%, MgSO_4_•7_2_O 0.06%, and PEG 6000 (5%) supplemented with minor elements (Kieser et al. 2000) were used to grow the *Streptomyces* strains; glucose, mannose, or lactose was added at a final concentration of 0.5% to evaluate antibiotic production, residual glucose, growth, and pH values. *E. coli DH5*α and LB broth (tryptone 10 g, yeast extract 5 g, NaCl 5 g/l) were used for standard cloning procedures, and the *E. coli* JM110 strain was used to obtain non-methylated DNA for transformation of the *Streptomyces* strains. *E. coli* and *Streptomyces* strains were generally incubated at 37 °C and 200 rpm and 29 °C and 300 rpm, respectively. Mannitol Soy flour Agar (MS) medium (mannitol 2%, soy flour 2%, agar 2%, MgCl_2_ 10 mM) was used for strain sporulation. For protoplast recovery and antibiotic production, R2 medium plates (sucrose 10.3%, K_2_SO_4_ 0.025%, MgCl_2_•6H_2_O 1.012%, glucose 1%, Casamino acids 0.01%, agar 0.01%) supplemented as described in Kieser et al. (2000) were used. The 2 × YT medium (tryptone 1.6%, yeast extract 1%, NaCl 0.5%) was used for spore pre-germination. Apramycin (50 µg/ml), kanamycin (50 µg/ml), and chloramphenicol (25 µg/ml) were used as selection antibiotics when necessary. All strains and plasmids used and generated in this work are summarized in Table S1, and primers previously reported from other works and those designed here are listed in Table S2.

### In silico analysis

The nucleotide and amino acid sequences of GarR/GarS from *S*. *coelicolor* were obtained from StrepDB (https://strepdb.streptomyces.org.uk). The operon prediction was performed by introducing the nucleotide sequence of the *S. coelicolor* M145 genome into the Operon-mapper (https://biocomputo.ibt.unam.mx/operon_mapper/) web server (Taboada et al. 2018). The SnapGene Viewer software was used to create and edit gene maps. InterPro EMBL-EBI (Paysan-Lafosse et al. 2022) and ScanProsite (De Castro et al. 2006) web tools (https://www.ebi.ac.uk/interpro/ and https://prosite.expasy.org) were used to predict protein domains. To predict the transmembrane domains for the SHK GarS the DeepTMHMM—1.0.24 (https://dtu.biolib.com/app/DeepTMHMM/run) platform was used (Hallgren et al. 2022). A reciprocal BLAST was performed in the Actinoblast web server (https://streptomyces.org.uk/actinoblast/) (Chandra and Chater 2014). The intrinsically disordered region (IDR) structure was predicted using the IntFOLD Integrated Protein Structure and Function Prediction Server version 7.0 (McGuffinet al. 2023). The proteins GarR/GarS models were generated using AlphaFold (Jumper et al. 2021) and edited using ChimeraX 1.6.1 software for MacOS (Pettersen et al. 2021). STRING version 12 was used (https://string-db.org) for protein–protein interaction (PPI) prediction (Szklarczyk et al. 2023); all were default settings, and the minimum interaction score was set to 0.700. The motif prediction was performed according to the method described by Anderssen et al. (2022), obtaining the sequence from − 400 to + 50 pb length upstream of the *sco6163* gene from *S. coelicolor* M145 and from 25 orthologous species of the Kyoto Encyclopedia of Genes and Genomes (KEGG), with a minimum SW score of 1500 and an identity above 0.78 (Table S3). These were submitted to the MEME Suite 5.5.2 (https://meme-suite.org/meme/) (Bailey et al. 2015) to find common motifs within the sequences using the default parameters and modify the model of sequences to 1st order and the maximum width of the motif to 30 nucleotides. Next, the three best-scoring motifs were compared to − 400 to + 50 pb upstream sequences of each of the genes within the complete *S. coelicolor* M145 genome uploaded to the MAST (Motif Alignment & Search Tool) to identify all genes containing similar motifs utilizing the default parameters. The description and function for each gene were assigned according to the Clusters of Orthologous Groups of Proteins (COGs) database.

### Generation of mutant strains

The standard procedures were performed as described in Gust et al. (2003). The primers were designed to amplify the apramycin resistance cassette by adding 39 nucleotides of the target genes to the ends (Table S2). These products were transformed into *E. coli* BW25113/pIJ790 with the cosmid St1A9 for homologous recombination. The recombinant cosmid was transformed into *E. coli* ET12567/pUZ8002 and conjugated into *S. coelicolor* M145 to disrupt the respective genes. Clones were selected with MS plates supplemented with apramycin (50 µg/ml). Two single and double mutant strains were generated: *∆garR*, *∆garS*, and *∆garR/∆garS*. To verify the correct deletion, the mutants were sequenced and probed by PCR with a set of primers (fwd6162shIn, rv6162shOut, fwd6163shIn, and fwd616shOut) that hybridized within the apramycin resistance cassette and an adjacent chromosomal region (Fig. S1).

### Complementation of mutant strains

The *garR* and *garS* genes were amplified by PCR using *S. coelicolor* DNA as a template with the *XbaIgarR_F—HindIIIgarR_R* and *XbaIgarS_F—HindIIIgarS_R* primers, respectively. The product of each set was cloned into the pKU1021 vector with the restriction enzymes *Hin*dIII and *Xba*I (NEB®). PCR-synthesized *3xFLAG* gene sequence with the self-hybridization of the primer set *3xFLAG_F* and *3xFLAG_R* designed to contain the optimized codon sequence for *Streptomyces* was cloned in frame with the *garR* and *garS* genes at the N-terminal region using *Nde*I and *Xba*I (NEB®) restriction enzymes. Finally, since the cloning of the *3xFLAG* sequence was done in the N-terminal region of each gene and to have these genes under the control of their promoter, a region located 400 bp upstream of the *garS* gene was amplified with the *F_Prom* and *R_Prom* primers and cloned into *Eco*RV and *Nde*I sites to replace the *S. avermitilis* promoter P*rpsJ* and to eliminate the kanamycin resistance cassette of the pKU1021 backbone. The resulting constructions, named pKU-*P3X-garR* and pKU-*P3X-garS*, were transformed into non-methylating *E. coli* JM110 for subsequent plasmid isolation and PEG-mediated transformation into mutant strains (Kieser et al. 2000). Transformants were selected by recovering protoplasts in R2 medium and re-culturing them in MS medium for sporulation, both with the addition of kanamycin (50 µg/ml). Complementation was verified by PCR detection of vector integration, sequencing, and expression detection of the tagged proteins by western blot analysis using a primary anti-DDDDK tag monoclonal antibody [FG4R] (GeneTex) and a secondary alkaline-phosphate-labeled anti-mouse immunoglobulin G antibody (rabbit ZyMAX™ ThermoScientific®).

### Evaluation of residual glucose, dry weight, and production of ACT and RED

The WT, mutants, and complemented strains were evaluated using standard procedures for growth (dry weight), residual glucose, ACT, and RED production. Dry weight was measured by vacuum-filtering 10 ml of mycelium onto a WhatmanTM 1 circular filter paper, which had been previously dried and weighed. The filter was dried with the sample to calculate the weight. The D-glucose GOD-POD (Spinreact) colorimetric method was used to determine residual glucose according to the manufacturer’s recommendations. ACT and RED were measured spectrophotometrically. For ACT, 0.5 ml of mycelium was collected, 0.5 ml of 3N KOH was added and incubated overnight at 4 °C with gentle agitation, and the absorbance was measured with a spectrophotometer at a wavelength of 640 nm. For RED, 1 ml of mycelium was collected and centrifuged, and the pellet was resuspended in 1 ml of 1 N acidified methanol (pH 2.1). After overnight incubation at 4 °C with gentle agitation, the absorbance was measured at a wavelength of 530 nm. All experimental data were obtained from triplicates simultaneously at each time point and from two independent experiments.

### RNA extraction and RT-qPCR assays

Samples from bacteria growth on NMMP supplemented with 0.5% glucose were harvested (at 24, 48, 72, 96, and 120 h). The extraction protocol was performed according to the Qiagen RNeasy mini kit instructions adapted from ActinoBase (Feeney et al. 2022) with some modifications.A total of 300 mg of mycelia were harvested, and 200 µl of TE buffer (20 µg/ml of proteinase K and 25 mg/ml of lysozyme) were added and mixed vigorously by vortexing. Immediately thereafter, 2.5 volumes of RNA Protect (Qiagen®) were added, mixed by vortexing, and incubated for 10 min at room temperature with constant agitation. Cells were lysed by the addition of 700 µl of RLT buffer supplemented with 1% mercaptoethanol and centrifuged at room temperature (10,000 rpm/10 min). The supernatant was recollected, purified with phenol/chloroform (1:1) centrifugated at 10,000 rpm/10 min, and finally precipitated with 50% volume of absolute ethanol. The RNeasy Mini Kit protocol was then followed, from transferring the samples to a column provided with the kit to elution with RNAse-free water. RNA sample integrity was assessed by quantification in Nanodrop One (ThermoScietific®) and visualized by electrophoresis on denaturing agarose gels. The samples were stored at − 80 °C until used. RNA samples were treated to remove DNA according to the manufacturer’s recommendations for Ambion™ DNase I (Thermo Scientific™). cDNA was prepared from 500 ng of RNA using random hexamer primers, dNTPs, RiboLock RNase inhibitor from Thermo Scientific™, and reverse transcriptase SuperScript II (Invitrogen). The primers for detection of *actII-ORF4*, *redZ*, *redD*, *garR*, and *hrdB* transcripts by qPCR experiments are listed in Table S4 and were performed using Maxima SYBR Green/ROX qPCR Master Mix (Thermo Scientific™) with the cycling parameters recommended by the manufacturer (initial denaturation 95 °C for 10 min, 40 cycles of denaturation 95 °C for 15 s, and annealing/extension for 60 s) in the Rotor-Gene Q real-time cycler (Qiagen®). For each reaction of 25 µl, 50 ng of retrotranscribed RNA was used as a template with 0.5 µM of the final concentration of each primer. The relative number of times gene expression of the samples was determined by comparing the mutant strain ∆garR with the M145 strain as control and calculated using the 2^−∆∆Ct^ method. Transcript levels were normalized to the sigma factor *hrdB* gene (Romero-Rodríguez et al. 2016b). For absolute quantification, a standard curve was generated using the pGEM-*garR* vector. Data from all qPCR experiments were obtained from two biological samples and three technical replicates. To detect the *garR*/*garS* gene transcript, RNA was treated with DNAse I, cDNA was generated as described above, and the target DNA was amplified by PCR using F transcript and R transcript primers.

## Results

### Genetic context of the TCS genes

**Fig. 1:**
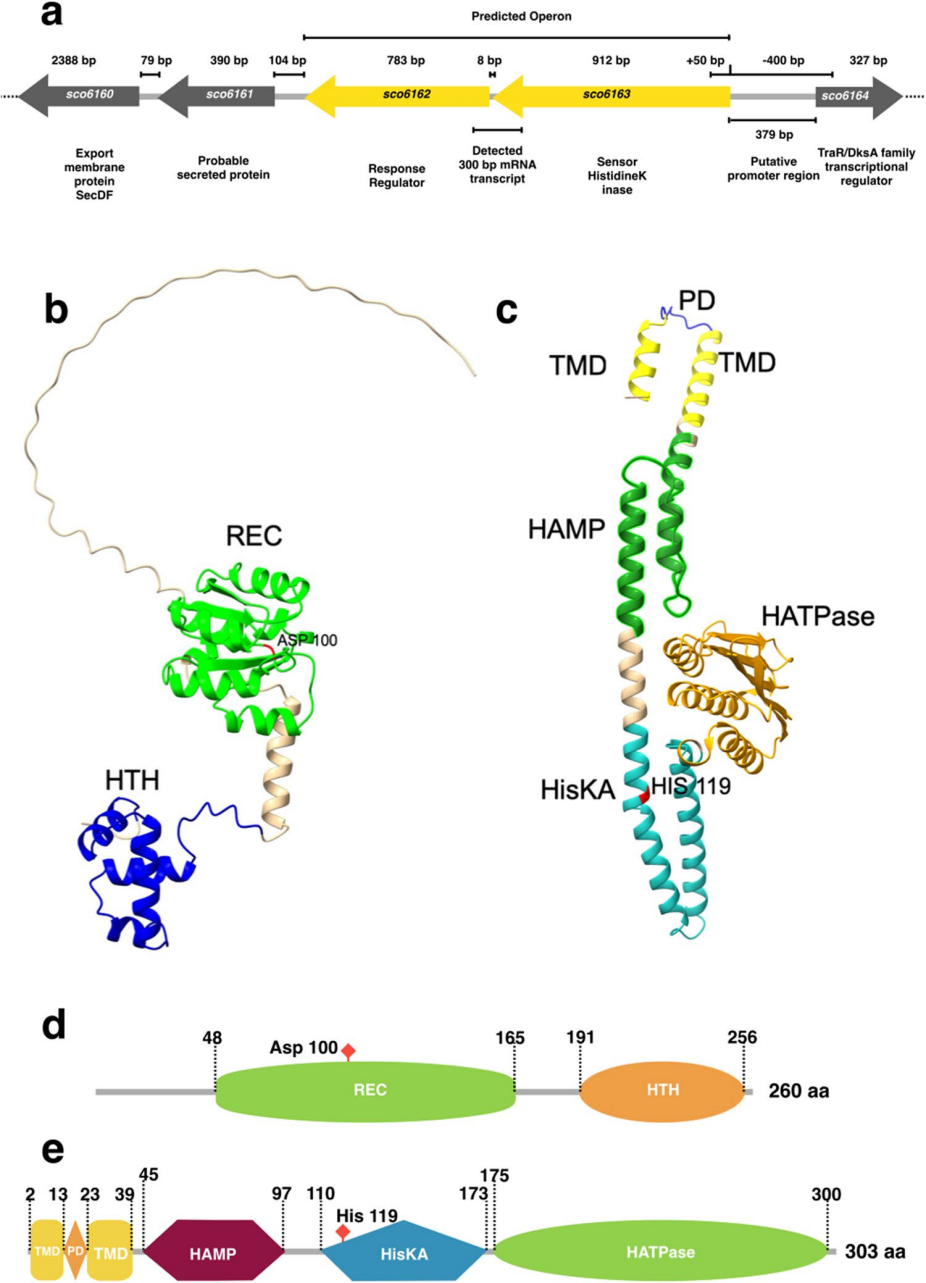
Genomic context of the two-component system coded in minus strand of the *S. coelicolor* chromosome. **a** The response regulator (RR) encoded by *garR* and the sensor histidine kinase (SHK) encoded by *garS* are represented with yellow arrows. Chromosome down- and upstream-located genes are gray-colored arrows, and the length of each gene, name, and probably coded proteins are presented. The length of the intergenic regions, operon prediction, putative promoter region, and the experimental mRNA transcript detected by RT-PCR are delimited by solid black lines. Dotted lines indicate the chromosome continuation. In silico monomer prediction of the two-component system proteins. **b** GarR (response regulator) presents an N-terminal receiver domain (REC) and a classical helix-turn-helix (HTH) DNA-binding domain in the C-terminal. The probable phospho-acceptor aspartate residues are in the 54, 55, and 100 positions (Asp 54, 55, and 100). **c** GarS (sensor histidine kinase) presents the classical periplasmic domain (PD), the transmembrane domain (TMD), the transmitter domain of conformational changes (HAMP), the signal transduction (HisKA), and phosphorylating (HATPase) domains. Histidine on the 119 position (His 119) is predicted as the phosphorylated conserved residue. **d** and **e** Lineal representation of the domain arrangement in GarR and GarS proteins

We analyzed the synteny conservation of both genes to investigate the potential role of the TCS. Both genes of the TCS are encoded in the negative strand and located on the left arm of the chromosome of *S. coelicolor.* These genes are not in phase being in a different open-reading frame and separated by 8 bp; the garR gene (sco6162) of 783 bp is located at genomic coordinates 6,765,623–6,766,405 and is annotated as a putative LuxR family member. The *garS* gene (*sco6163*) of 912 bp is located at genomic coordinates 6,766,414–6,767,325 and is predicted to be a probable SHK. The TCS genes are located between a downstream-encoded putative-secreted protein (*sco6161*) at 104 bp and an upstream intergenic region of 379 bp containing the putative promoter region followed by a hypothetical protein (*sco6164*) encoded in the positive strand. However, despite their proximity, only the *garR/garS* genes are predicted to be co-transcribed by the Operon-mapper web server with only an 8 bp separation in between (Fig. 1a). This prediction was confirmed by an RT-PCR assay using a total RNA sample from the WT strain of *S. coelicolor* M145. A pair of primers were designed and used to detect a 300 pb, including the 8-bp intergenic region between both genes (Fig. 1a). The amplified PCR product was sequenced, confirming the presence of a transcript containing both genes (Fig. S2).

A search with the reciprocal BLASTP available in the Actinoblast database suggested that the garR/*garS* TCS is not widespread in the suborder *Streptomycineae*. The orthologs of both genes are reported only in *Streptomyces lividans* TK24, *Streptomyces viridochromogenes* DSM 40736, *Streptomyces scabies* 87.22, and *Streptomyces pristinaespiralis* ATCC 25486. Orthologs of the RR alone exist only in *Streptomyces griseus* subsp. *griseus* NBRC, *Streptomyces hygroscopicus* ATCC 56356, and *Streptomyces roseosporus* NRRL 15998. In contrast, the gene encoding the SHK alone is present only in *S. griseoflavus* Tu4000.

### Protein modeling

To identify if the TCS proteins had the elements to sense and transduce signals to elicit a response, we performed a protein-folding prediction with Alphafold and a protein domain prediction with InterPro, ScanProsite, DeepTMHMM, and IntFOLD tools. The 260-amino-acid (aa) GarR sequence (28.31 kDa) is predicted to have an N-terminal REC domain (48–165 residues) which receives the phosphate from the SHK in a conserved aspartate and a C-terminal LuxR family domain (191–256 residues) that has a helix-turn-helix (HTH) arrangement for DNA-binding activity. The phosphoryl aspartate was predicted to be at the aa position 100 (Fig. 1b and d). In the generated model, a long amino acid tail of 48 residues was analyzed, and an IDR of 44 residues was predicted. These IDRs have been associated with promiscuous interactions when present in proteins with DNA regulatory activity, but they may also be necessary to localize the DNA-binding domains (Ferrie et al. 2022). On the other hand, the 303-amino-acid GarS protein (32.83 kDa) was analyzed by InterPro Scan (Fig. 1c and e) and predicted to have a classical HAMP domain (45–97 residues) that transmits the conformational changes from the sensor domain to the cytoplasm. A HATPase domain between the residues 175 and 300 is responsible for phosphorylating a conserved histidine residue in the HisKA signal transduction domain of residues 110–173. Amino acid 119 was predicted to be the conserved phospho-histidine residue. The TMHMM tool predicts a topology that contains two transmembrane domains (TMD) in regions 2–13 and 23–39 and a periplasmic domain (PD) of 9 aa between the two TMD (Fig. 1c and e), which is unlikely to be a sensor domain. Interestingly, an aa sequence alignment of the GarR/GarS and SCO5784/SCO5785 TCSs showed a high percentage of identity between them—78% for GarR and SCO5785 and 59% for GarS and SCO5784. The *sco5784*/*sco5785* genes are located on the positive chromosome strand of *S. coelicolor*. This TCS has been shown to affect antibiotic biosynthesis and enhance the production of some secretory proteins (Uguru et al. 2005), and both proteins have a similar domain organization (not shown). Similarly, GarS and SCO6424 share 59% identity (not shown).

### In silico protein interaction of GarR/GarS suggests a role in antibiotic biosynthesis

**Fig. 2:**
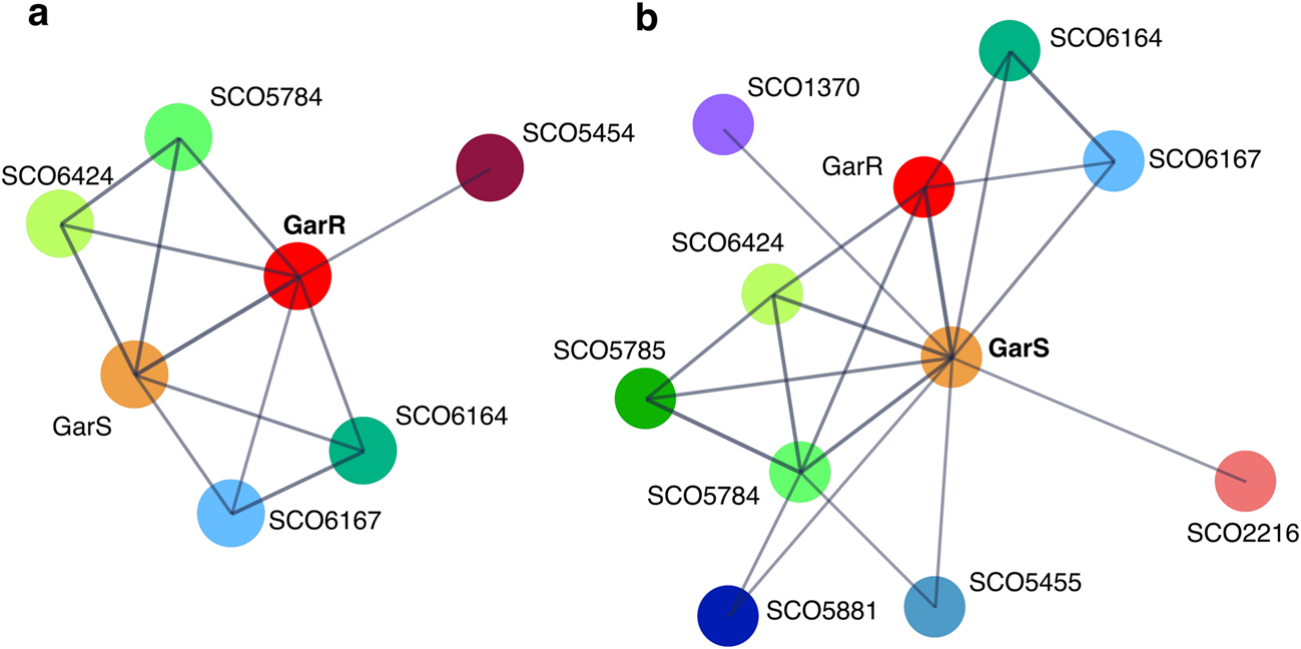
Predicted interactomes by STRING of the proteins of the two-component system GarR (**a**) and GarS (**b**). The colored circles represent proteins with predicted interaction, and the line intensity is directly related to the confidence in the prediction

To assess the potential involvement of the TCS in antibiotic biosynthesis and regulation, we constructed two PPI networks with the STRING server. The individual and combined confidence values for each interaction in the network are listed in Tables S5 and S6. The PPI network for GarR resulted in 7 nodes, 12 edges, an average node degree of 3.43, a PPI enrichment *p*-value of 0.0208, and a confidence score average of 0.820 between GarR and other proteins. Here, in addition to the interaction with GarS, the RR interacts with three other TCS-SHKs (SCO5454, SCO5784, SCO6424). It also appears to be associated with two other putative proteins, a proline-rich membrane protein (SCO6167) and an unknown hypothetical protein (SCO6164) (Fig. 2a). The PPI network for GarS resulted in 11 nodes, 20 edges, an average node degree of 3.64, a PPI enrichment *p*-value of 0.00392, and an average confidence score of 0.805 between GarS and other proteins. As with the previous prediction, the interaction with GarR appeared and was also predicted to interact with four RRs belonging to different TCSs. This is the case for SCO1370, SCO5785, SCO5455, and SCO2216. Interestingly, the interaction with RedZ (SCO5881), considered an atypical RR positive regulator of the RedD cluster biosynthesis (Sánchez de la Nieta et al. 2022), was also predicted (Fig. 2b).

### Putative genes regulated by GarR suggest the interaction with other TCSs and transcriptional regulators

**Fig. 3:**
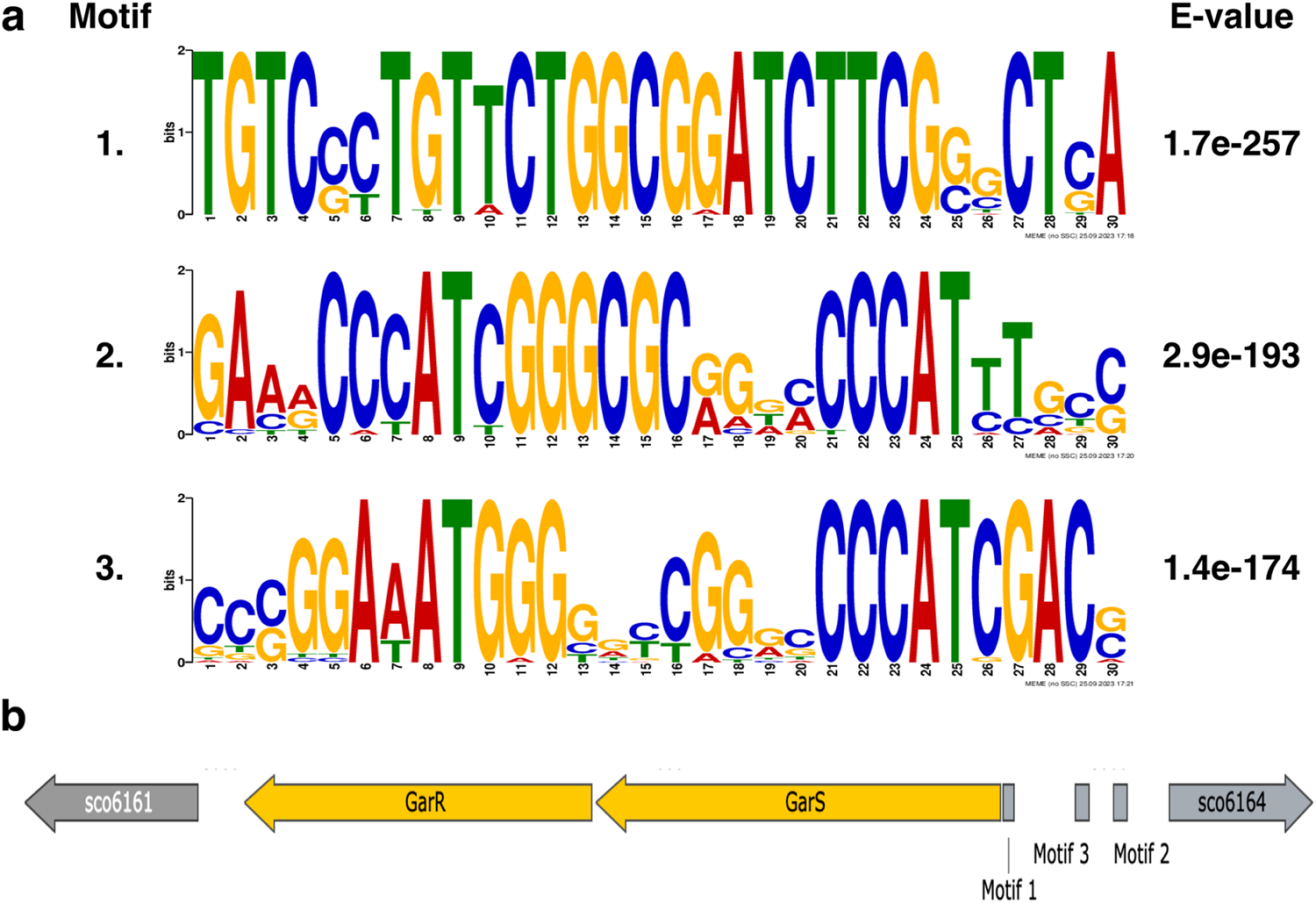
**a** The three 30-pb-long motifs generated by the MEME Suite 5.5.2. The statistical significance of each motive is presented in the right column. **b** Graphical distribution of the three motifs in the upstream region of *garR*/*garS*. All three motifs are in the coding strand

An in silico analysis was performed to infer the possible target genes of GarR. For this purpose, the region from − 400 to + 50 upstream was used as a probe to search for other similar promoter regions. We searched for conserved motifs postulated as possible GarR binding sites from the sequences identical to the above region. We selected three predicted motifs (Fig. 3) that were analyzed for likely genes in the chromosome that would share those sequences in their upstream − 400 to + 50 regions. In the resulting predictions and from the COGs classification (Table S7 and Fig. S3), we observed that the three motifs were found in the upstream areas of genes that are involved in the signal transduction mechanisms (TCSs). Motif 2 was found in the upstream region of the *sco1370* gene and motif 3 was in the upstream region of the *sco6424* and *sco5784* genes, encoding one RR and two SHKs, respectively (Table 1). *sco6424* and *sco5784* appeared in both interactome predictions, but *sco1370* only appeared in the GarS interactome, supporting the likely interaction between these TCSs.

**Table 1:**
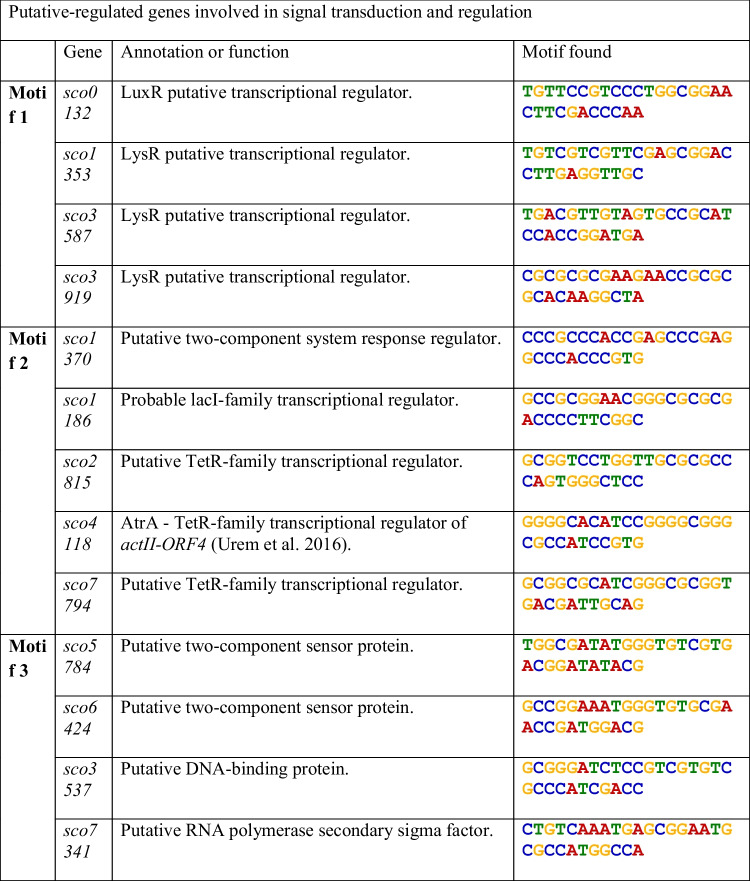
List of the putative-regulated genes involved in signal transduction and regulation

Sequences of motifs 1, 2, and 3 were found in the putative promoter regions of the listed regulatory genes

On the other hand, sequences of motifs 1 and 2 were found in the upstream regions of genes encoding for additional regulatory proteins. For motif 1, *sco0132* (LuxR family), *sco1353*, *sco3587*, and *sco3919* (LysR family). For motif 2, *sco1186* (LacI family), *sco2815*, *sco4118*, and *sco7794* (TetR family) (Table 1). Notably, the *sco4118* gene encodes for AtrA, a positive regulator of ACT targeting *actII-ORF4* (Uguru et al. 2005). For motif 3, the putative DNA-binding protein encoded by *sco3537* contains an HTH domain and a putative RNA polymerase sigma factor encoded by *sco7341* (Table 1). Other putative-regulated genes by GarR in each motif are related to carbohydrate transport and metabolism, which are typically involved in regulating growth, development, and secondary metabolite production (Ruiz-Villafán et al. 2021). Despite that only motif 1 was found in the upstream region of one gene involved in secondary metabolite biosynthesis (*sco6998*), and that none of the genes within the biosynthetic clusters of ACT or RED were predicted as targets, the regulation of other TCSs and TFs, including a regulator of ACT, may indicate that secondary metabolites regulation is exerted indirectly through other pathways.

### GarR/GarS affects ACT and RED production

**Fig. 4:**
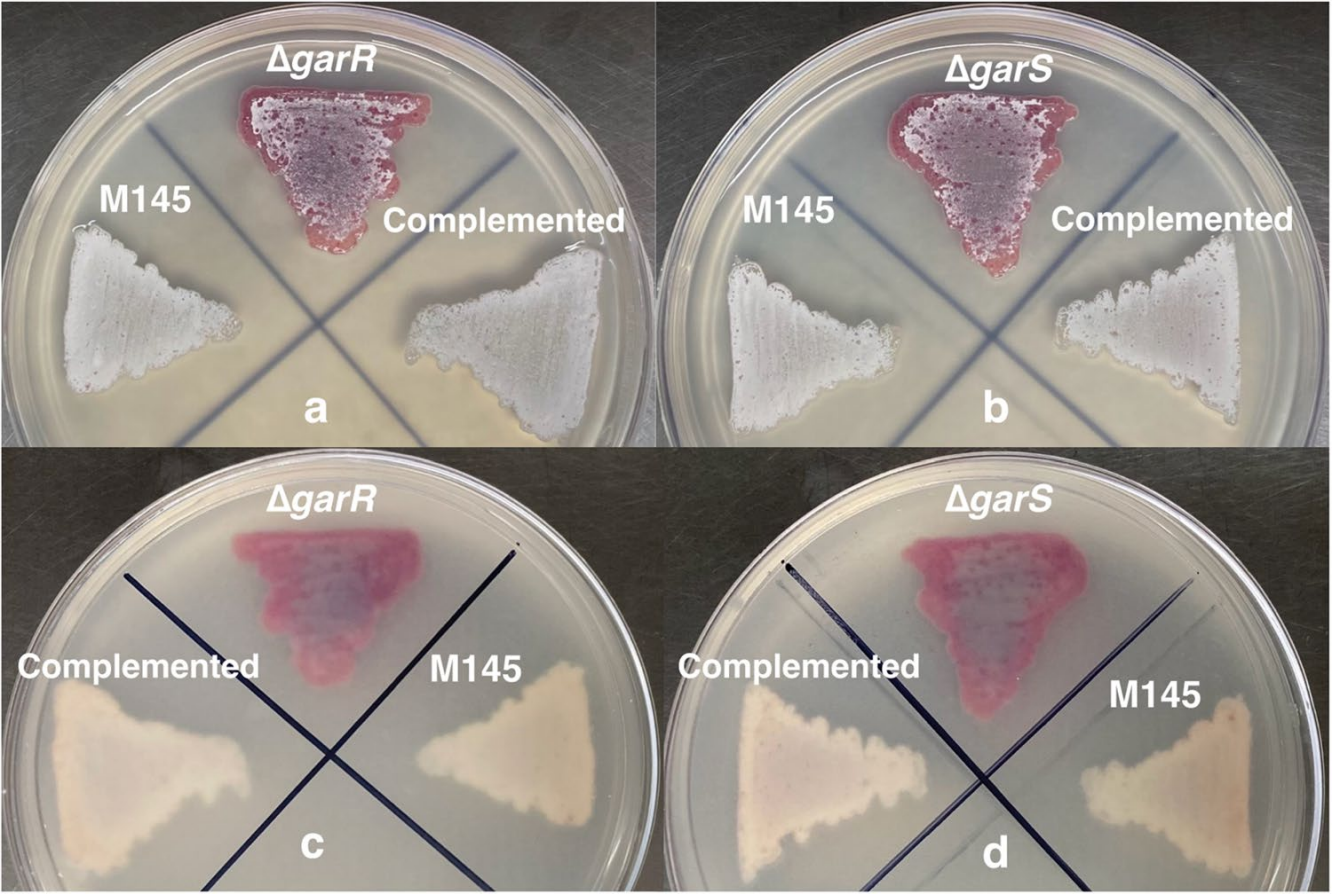
Front (**a** and **b**) and back (**c** and **d**) of the growth spores of the wild-type *S. coelicolor* M145, its derived mutants *∆garR* (**a** and **c**) and *∆garS* (**b** and **d**), and their respective complemented strains (*∆garR-*pKU-P3X-*garR* and *∆garS*-pKU-P3X-*garS*) in NMMP media supplemented with 0.5% of glucose after 48 h at 29 °C

**Fig. 5:**
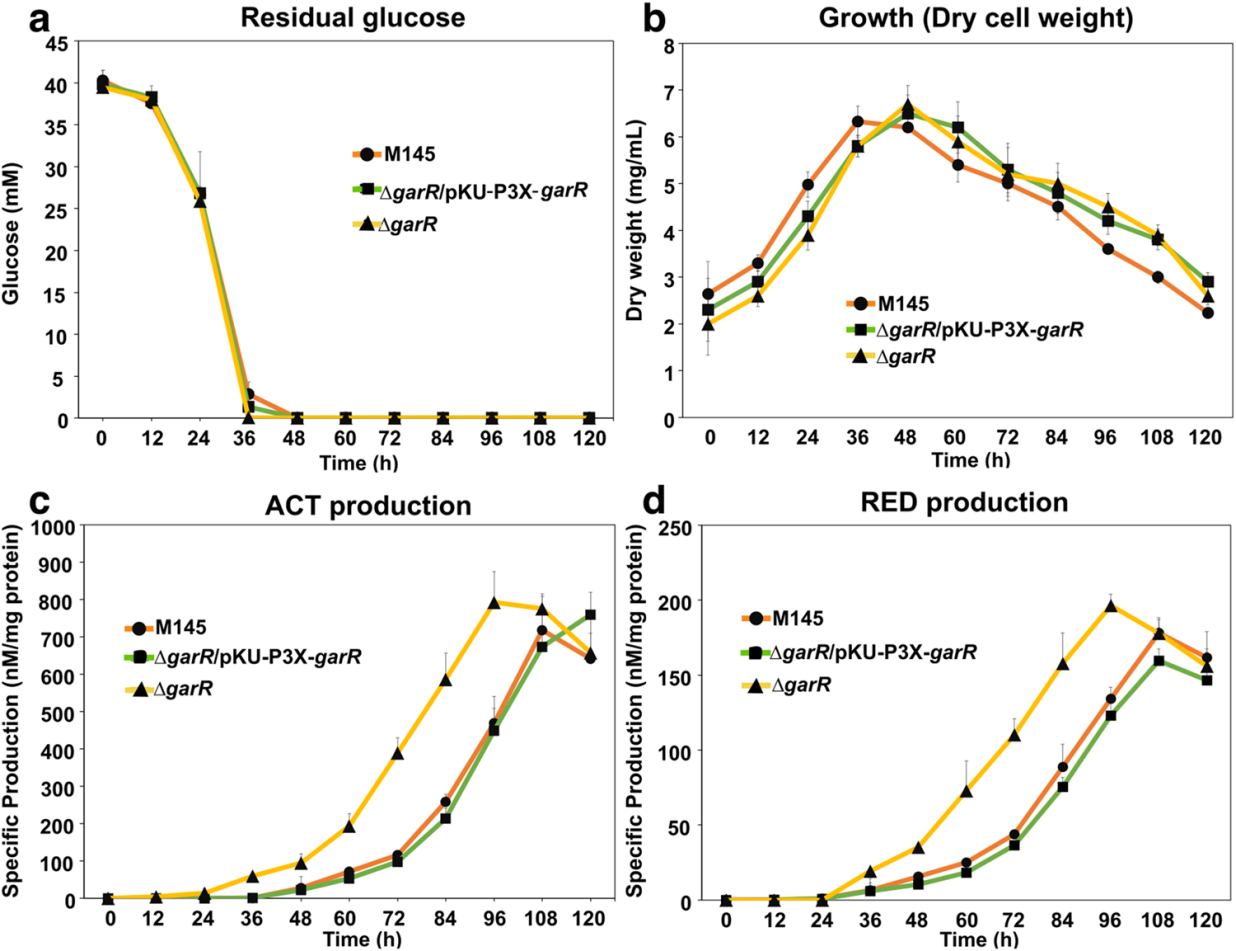
Evaluation of the wild-type *S. coelicolor* M145 (•), the mutant complemented with the pKU-*P3X-garR* vector integrated (◼), and the *∆garR* mutant (▲) *Streptomyces* strains grown in NMMP supplemented with 0.5% glucose during 120 h (horizontal axis). The vertical axis indicates **a** concentration of the residual glucose in the medium, **b** growth measured by dry cell weight, **c** specific actinorhodin (ACT) production, and **d** specific undecylprodigiosin (RED) production

**Fig. 6:**
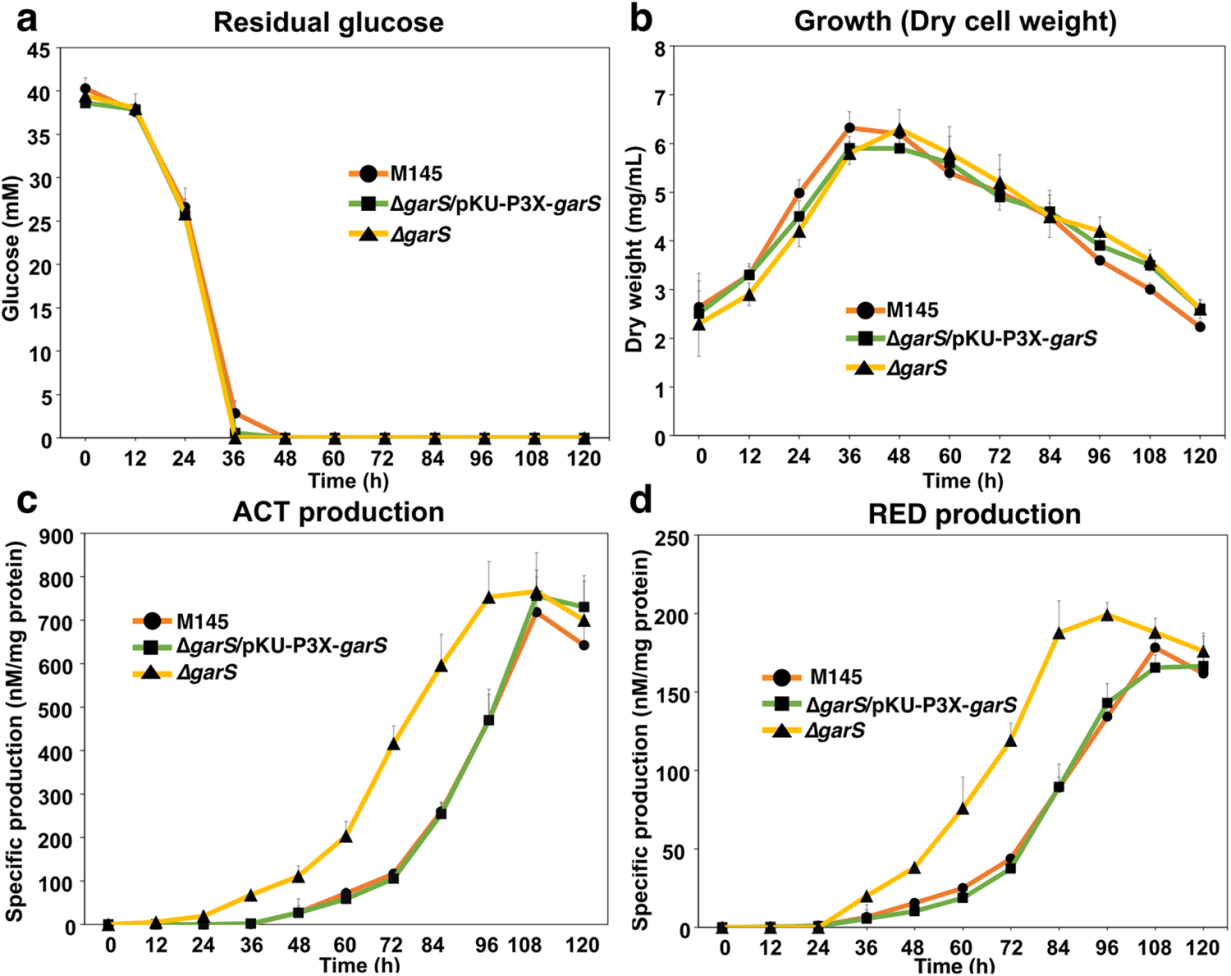
Evaluation of the wild-type *S. coelicolor* M145 (•), the mutant complemented with pKU-*P3X-garS* vector integrated (◼), and *∆garS* mutant (▲) *Streptomyces* strains grown in NMMP supplemented with 0.5% of glucose during 120 h (horizontal axis). The vertical axis indicates **a** concentration of the residual glucose in the medium, **b** growth measured by dry weight, **c** specific actinorhodin (ACT) production, and **d** specific undecylprodigiosin (RED) production

We had previously observed that GarR/GarS was transcriptionally repressed by glucose in the medium compared to a strain grown under non-repressive conditions (Romero-Rodríguez et al. 2016b). Knowing that in *S. coelicolor*, the pigmented antibiotics ACT and RED are repressed by glucose in a minimal liquid medium (NMMP) supplemented with this carbon source (Romero-Rodríguez et al. 2016a), and with the information obtained from the in silico protein and gene interactions previously shown, we decided to evaluate the influence of GarR/GarS under these conditions. For this purpose, we generated two single- and one double-deleted mutant strains (*∆garR*, *∆garS*, and *∆garR*/*∆garS*). PCR and DNA sequencing confirmed the deletions by detecting the presence of the apramycin resistance cassette and the absence of the corresponding genes. These null mutants were evaluated in the R2 solid medium (Fig. 4), exhibiting a clear overproduction of pigmented antibiotics compared to the WT M145 strain. This result was corroborated when the mutants were grown in an NMMP medium supplemented with glucose. As shown in Figs. 5 and 6c and d, the production of both pigmented antibiotics was derepressed even in the presence of glucose when one or both genes of the TCS were absent compared to the WT M145 strain. To determine if this effect was due to the presence of glucose in the medium, all strains were evaluated in NMMP supplemented with mannose or lactose (0.5%) as the sole carbon source, and no significant differences in ACT and RED production were observed between all mutant strains and the control WT M145 strain (Fig. S4). To confirm that the TCS was responsible for the observed phenotype, the two single mutant strains were complemented by reintegrating the respective genes (both with the *3xFLAG* tag sequence fused at the N-terminus) into a vector containing the ΦC31 integrase system (pKU1021). Complementation was verified by PCR, sequencing, and Western blot analysis to detect the presence of the 3xFLAG-tagged proteins (Fig. S5), with predicted molecular weights of 31.3 kDa (GarR) and 35.81 kDa (GarS) when fused with the tag. These two strains were also evaluated for residual glucose, growth, and production of ACT and RED. Here, we observed that the production of ACT and RED was repressed upon restoration of TCS proteins (Fig. 4) and in the presence of glucose (Figs. 5 and 6c and d) without affecting growth and residual glucose concentration in the culture medium (Figs. 5 and 6a and b) The above results were obtained with no variations in the pH levels of the culture media of strains that were tested.

### GarR affects the transcription of ACT and RED regulators

**Fig. 7:**
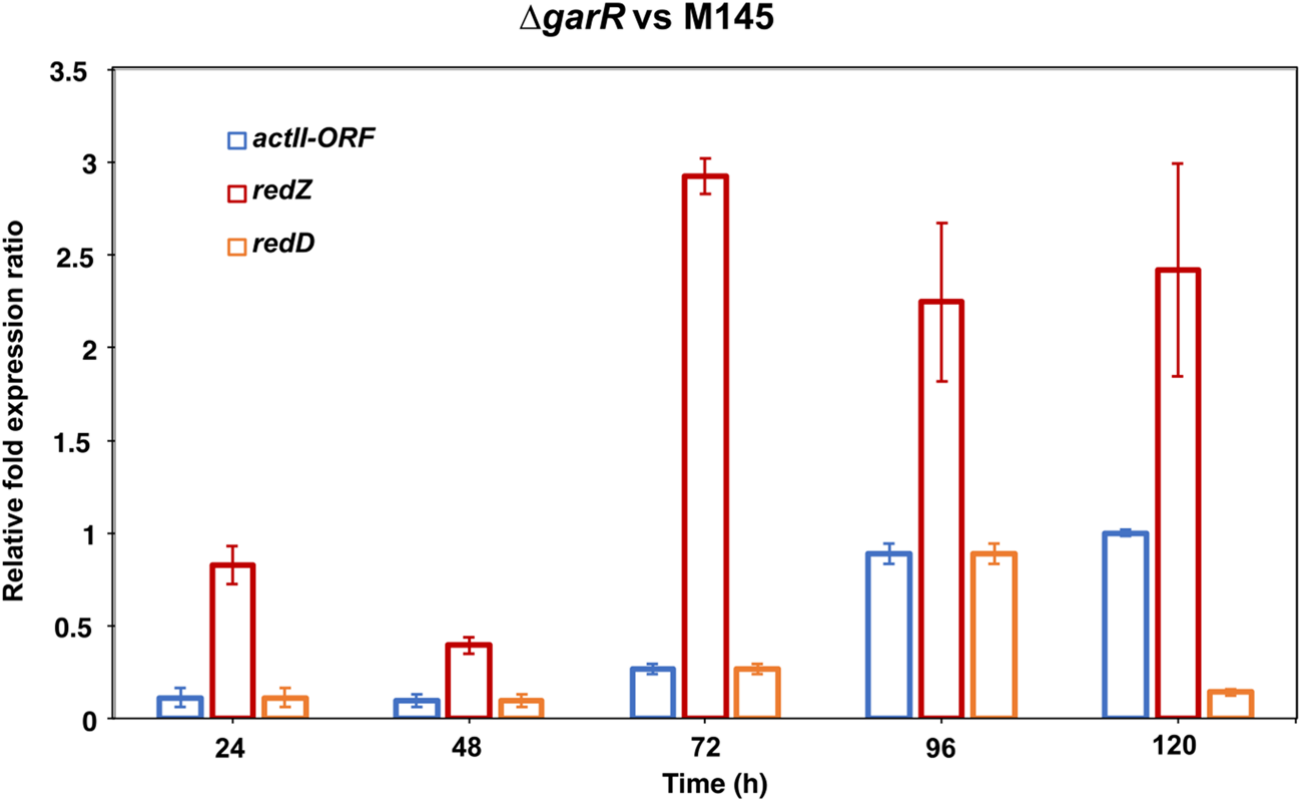
Relative expression (RT-qPCR) of *actII-ORF4* mRNA, *redZ*, and *redD* in wild-type strain *S. coelicolor* (M145) and mutant strain lacking the RR (∆*garR*) growth in NMMP supplemented with glucose (0.5%) harvested every 24 h during 120 h. Relative expression was normalized to the *hrdB* sigma factor gene. Data was obtained from two independent experimental samples with three technical replicates each, and the 2^−∆∆Ct^ method was applied to calculate the relative fold gene expression of the samples. Error bars represent the standard deviation

To gain insight into the likely regulation of both antibiotics, we assessed the relative mRNA expression of the master regulators for ACT (*actII-ORF4*) and RED (*redD* and *redZ*) using qPCR analysis of the mutant lacking the RR GarR under repressive conditions. As shown in Fig. 7, increased transcription of all three regulators was observed in the mutant compared to strain M145. This was consistent with the production of ACT and RED by the *∆garR* and WT M145 strains. Thus, the relative expression of *actII-ORF4* was increased by up to 80%, and the increase of *redZ* was even more pronounced with up to almost 200% compared to strain M145 (Fig. 7).

## Discussion

*Streptomyces* spp. is notable for producing many secondary metabolites, including more than 2/3 of clinically used antibiotics (Donald et al. 2022). The mechanisms these microorganisms utilize to regulate the production of those metabolites are complex and still not fully understood (Ruiz-Villafán et al. 2021). It is well-known that the mechanisms involved in the regulation are mediated by proteins capable of sensing and responding to different stimuli in the media, such as nitrogen, carbon, and phosphate sources, among others (Romero-Rodríguez et al. 2018). These mechanisms include the TCSs, which are integrated by two proteins, one capable of sensing a stimulus and the other capable of emitting the response according to the signal generally at the DNA level (Buschiazzo and Trajtenberg 2019). *Streptomyces* contain the highest number of TCSs, reflecting their need to adapt to the environment by tunning primary and secondary metabolism (Romero-Rodríguez et al. 2015; Arroyo-Pérez et al. 2019). Approaches to studying these proteins range from using bioinformatic tools to various experimental techniques that allow an understanding of the molecular mechanisms by which the TCS senses and responds to the stimulus (Cruz-Bautista et al. 2023).

In the present work, we carry out a general analysis of the TCS SCO6162/SCO6163, here renamed as GarR/GarS (Glucose-responsive antibiotic regulator), due to the observation that this TCS regulates antibiotic production in response to glucose. By in silico prediction analysis of protein interaction and transcriptional regulation, combined with qPCR studies of deleted and complemented GarR/GarS mutants, we had an insight into the negative regulation of ACT and RED production by GarR/GarS. We first applied a genomic context approach to obtain information about the likely role of GarR/GarS by predicting PPIs and visualizing their possible functional roles (Botas et al. 2022). We observed that two genes encoded in the downstream region of *garR/garS* (*sco6160* and *sco6161*) are a putative SecDF export protein (SCO6160) and a secreted protein (SCO6161), respectively (Fig. 1a). These genes appear unrelated to *garR/garS* at the transcriptional level. Furthermore, using the Operon Mapper tool, *sco6160* and *sco6161* do not seem to be part of the operon. However, these genes, including *garR/garS*, have been observed to be associated with cell envelope maintenance in an *S*. *coelicolor* mutant strain M145*sco5282*-D125G, which exhibits a dispersed growth phenotype when grown in liquid media (Arroyo-Pérez et al. 2019). A TraR/DksA C4-type zinc finger protein is putatively encoded by the gene located 379 bp upstream of *garR/garS* in the positive strand (*sco6164*) (Fig. 1a). However, this gene also seems unrelated to *garR/garS* at the transcriptional level. On the other hand, using RT-PCR assays, we experimentally confirmed that garR/*garS* genes are co-transcribed (Fig. S2).

Bioinformatic analysis of the gene products confirmed that GarR is an RR protein of 28.34-kDa size, and GarS is a SHK protein of 32.83 kDa. GarR is annotated as part of the LuxR family, identified as regulators of the secondary metabolism in *Streptomyces*, *which interact with other regulators involved in BGC* transcription (Zhang et al. 2022). GarR has two domains—a conserved N-terminal REC domain, responsible for receiving a phosphate group from SHK and presenting the classical five α-helices around five central ß-sheets (Gao et al. 2019). A conserved aspartate of the REC domain at the 100 amino acid position is responsible for receiving the signal from SHK (Fig. 1b and d). The second, less conserved C-terminal region presents a DNA-binding domain in a helix-turn-helix arrangement, classified as part of the NarL subfamily (Brunet et al. 2022). This domain interacts with the target DNA to develop the appropriate response. The generated model for the RR predicted an IDR of 44 amino acids in length; these structures are well-studied in eukaryotic cells, where they facilitate DNA-binding and PPIs, among other chemical processes (Ferrie et al. 2022). In prokaryotes, their function is less clear, and only a few IDRs function as cytoplasmic interaction sites with FtsZ and PopZ proteins. More recently, a sensing function responding to cell wall defects by RsgI–σI in *B. subtilis* has been reported for IDRs (Brunet et al. 2022). The presence of this IDR in the RR is attractive since it could play a role for GarR as a pathway regulator. The SHK is predicted to have the classical TM, HAMP, HisKA, and HATPase domain architecture (Fig. 1c and e). The two TMDs are interrupted by a short periplasmic domain of 9 amino acids, which is unlikely to contain a sensor domain and, therefore, may sense through the transmembrane segments (Cheung and Hendrickson 2010). A similar example occurs with proteins like DesK from *Bacillus subtilis*, which senses membrane fluidity in a temperature-dependent manner (Abriata et al. 2017), or SenS from *S. reticuli* and *S. coelicolor* which is a three-component system that senses by interacting with the HbpS protein that modulates autophosphorylation (Busche et al. 2016). The predicted conserved histidine, responsible for transducing the signal into the RR, was predicted to be at position 119 (Fig. 1c and e). These data predicted that the GarR/GarS is a classical TCS containing all the necessary domains to perform signal transduction and elicit a response at the transcriptional level.

The bacterial process, including the formation of secondary metabolites, is a highly regulated mechanism supported by various essential steps, including transcription and PPIs (Carro 2018; Bervoets and Charlier 2018). With this in mind, we performed two in silico predictions of the GarR and GarS PPIs with STRING and one of the putative genes regulated by GarR. The first result shows that this TCS will likely interact with other TCSs (Fig. 2), suggesting crosstalk between them. This phenomenon, where an SHK can activate a non-cognate RR and vice versa, is well-described. An RR can be phosphorylated by a non-cognate SHK, allowing the communication of different pathways with this redundancy (Agrawal et al. 2016). This explanation may be the case for the TCS SCO5784/SCO5785, which shows a high identity with GarR/GarS (SHKs, 58.86%; RRs, 77.52%) (Fig. 2). SCO5784/SCO5785 has been shown to affect the production of ACT and RED (Rozas et al. 2012). GarS and the SCO6424 SHK also show high identity (59.08%) and appear in both interactomes (Fig. 2). Together with the experimental data observed here, we can suggest a redundancy and/or a crosstalk interaction between them that could link ACT and RED regulation. Likewise, GarR was predicted to interact with the SCO5454 SHK, and GarS SHK with SCO1370, SCO5455, and SCO2216 RRs (Fig. 2), which have not yet been studied. The putative interaction between GarS and SCO5881 (RedZ) caught our attention since the latter is a positive regulator of RED by directly regulating the positive regulator RedD (Sánchez de la Nieta et al. 2022). In this context, RedZ is considered an atypical RR, which is not encoded next to a cognate SHK, and lacks the conserved structure required for phosphorylation, and its regulation is mediated by its end-product RED (Liu et al. 2013). STRING has been widely used to understand and predict PPIs; these interactions stem from computational prediction, knowledge transfer between organisms, and interactions aggregated from other (primary) databases. The interactions in STRING are derived from five main sources as follows: genomic context predictions, high-throughput laboratory experiments, conserved co-expression data, automated data mining, and previously deposited knowledge in databases (Szklarczyk et al. 2023). Predictions obtained here have, in general, a high confidence value and low *p*-values, indicating that the protein nodes are not random and that the observed edges are significant. These are solid predictions that can conduct future experiments to understand the pathways by which this TCS regulates antibiotics. The success in using these types of predictions has been widely reported (Fridlich et al. 2009; Thakur and Gauba 2024; Yan et al. 2023; Hanes et al. 2023). Since the interactome predictions made by STRING include functional and physical associations and coupled with the observed modification of both transcripts (redZ and redD) by qPCR, we can suggest that the regulation of RED production by GarR/GarS may be due to the involvement of the RedZ-RedD mini cascade pathway. It is worth mentioning that the TCS SCO5784/SCO5785 and the orphan SCO6424 SHK were also reported to be downregulated in transcriptomic experiments under repressive conditions (Romero-Rodríguez et al. 2016b), strengthening their likely relation.

To complement the likely regulatory pathways at the transcriptional level, we aim to predict the likely genes regulated by GarR. Motif prediction was based on the assumption that the TFs can be autoregulated (*cis-acting*) by a motif upstream of their coding region, an approach successfully used with other regulatory family proteins (Anderssen et al. 2022). Based on this methodology, we made some modifications. We selected three motifs with the highest *E*-values that were searched in all the upstream regions (− 400 to + 50) of each gene in the *S. coelicolor* chromosome (Fig. 3). Among all predicted genes in the three motifs, we observed that their functionality includes important processes like transport and metabolism of amino acids and carbohydrates, transcription, regulatory proteins, and signal transduction mechanisms among others (Fig. S3). As in the PPI predictions, the genes for the SHKs *sco5784* and *sco6424* appeared as likely regulated genes, as well as an RR encoded by *sco1370* which only appears in the SHK GarS interactome (Table 1). Of the remaining predicted genes, it is important to note that some were annotated to encode for regulatory proteins of different families, and among these, *sco4118* was highlighted for being reported as the positive regulator of ACT (AtrA), which directly regulates the inner cluster regulator *actII-ORF4* (Uguru et al. 2005). Orthologs of AtrA also directly impact the biosynthesis of other antibiotics, like avermectin in *S. avermitilis* (Liu et al. 2019) and lincomycin in *S. lincolnensis* (Wu et al. 2023). All these data for the in silico predictions let us conclude that GarR/GarS plays an important role in ACT and RED biosynthesis. Therefore, we decided to support it with some experimental data.

Since GarR/GarS is overexpressed in the presence of glucose (Romero-Rodríguez et al. 2016b) and considering its likely relationship with the biosynthesis of the two pigmented antibiotics predicted here, we aimed to study the effect of the TCS on the ACT and RED production under repressive conditions (glucose 0.5%). Therefore, we constructed three mutants lacking one or both TCS genes that were successfully probed by PCR and sequencing (Fig. S1). These strains showed an increased production of both antibiotics compared to the WT M145 strain, even under repressive conditions, suggesting a repressive effect on ACT and RED production (Figs. 5 and 6c and d). Later, we evaluated all strains in the presence of mannose or lactose (0.5%), where we observed no difference in antibiotic production between all strains (Fig. S4), suggesting that this effect is due to the presence of glucose in the medium. To gain more evidence for the impact of GarR/GarS, we later complemented the single mutant strains with the respective genes fused to an optimized sequence of the *3xFLAG* gene for *Streptomyces* under the control of its promoter. The reversion of ACT and RED production to the WT M145 levels was shown to be due to the presence of GarR/GarS (Figs. 5 and 6c and d). In all evaluations of both complemented strains, growth (dry weight), residual glucose (Figs. 5 and 6a and b), and pH were not affected, suggesting again that this TCS is involved in the biosynthesis of the ACT and RED. With this result, we also demonstrated that the fusion of the 3xFLAG tag protein to the N-terminal of both TCS proteins (Fig. S5) did not interfere with their expression and function. Since Δ*garR* and Δ*garS* mutants overproduced ACT and RED, we evaluated whether the inner cluster regulators of both antibiotics were modified at the transcriptional level when these strains were grown under repressive conditions compared to the WT M145 strain. As expected, the three regulators, *actII-ORF4* (ACT), *redZ*, and *redD* (RED), were upregulated (Fig. 7). The overexpression of the *redZ* gene, which in turn activates the *redD* gene to initiate RED biosynthesis, was an anticipated result since *redZ* is expressed earlier than the other RED cluster genes to initiate the RED biosynthesis, just at the beginning of the exponential growth phase (Huang et al. 2001). These results coincide with those observed in the spectrophotometric antibiotic determinations, supporting the idea that the regulation of ACT and RED is affected at the transcriptional level and, at some point, involves these pathways.

**Fig. 8:**
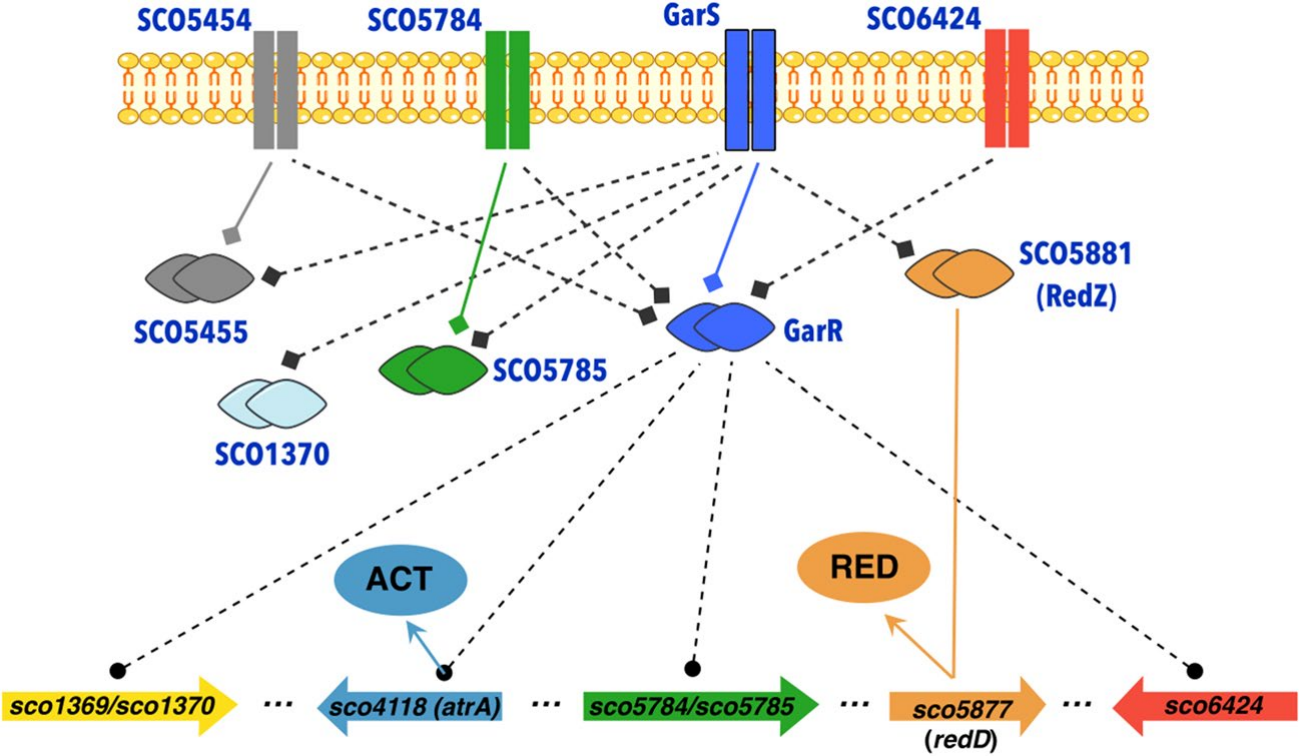
Presumptive model of the possible regulatory pathways of actinorhodin (ACT) and undecylprodigiosin (RED) by GarR/GarS and their interaction with other two-component systems. Black dashed lines ending in diamonds indicate inferred functional associations as predicted by STRING. Dashed black lines ending in a circle show their possible transcriptional targets as predicted by the MEME suite. Solid-colored lines ending in an arrow display known regulatory pathways. The other putative two-component genes are *sco5454*/*sco5455* (SHK/RR), *sco5784*/*sco5785* (SHK/RR), *sco1369*/*sco1370* (SHK/RR), and *sco6424* (SHK)

TCSs in *Streptomyces* are abundant and essential for adaptation, yet, only a few have been characterized. It is important to continue their study, as this knowledge would allow metabolic engineering strategies to improve the production of secondary metabolites and discover new ones (Cruz-Bautista et al. 2023). This work provides an overview of the TCS GarR/GarS from *S. coelicolor* by combining bioinformatic tools and experimental methods. Combining all these data, we can conclude that GarR/GarS is a negative regulator of the ACT and RED production under repressive conditions with 0.5% glucose in the medium. Also, this regulation is at some point mediated by modifying the transcription of the internal master regulators of both antibiotics, likely AtrA for ACT and RedZ for RED. With the in silico predictions, we generated a putative model (Fig. 8) to understand and guide the search for the antibiotic pathways regulated by GarR/GarS.

The performance of bioinformatic studies on the protein structure and conserved residues would give valuable information for future experiments to explain the molecular mechanisms by which GarR/GarS senses and transmits the signal to the putative-regulated genes. Finally, even though this work focused on the regulation of the two pigmented antibiotics produced by the *S. coelicolor* model and the predictions carried out here did not detect the direct regulation of other BGCs, the indirect regulation by other TCSs and/or TFs cannot be discarded.

## Supplementary Information

Below is the link to the electronic supplementary material.

Supplementary file1 (PDF 740 KB)

## Data Availability

All data generated or analyzed during this study are included in this manuscript and its supplementary information files. Requests for any additional information can be made to the corresponding author.
