# Supplementary material for: An overview of the two-component system GarR/GarS role on antibiotic production in Streptomyces coelicolor
Source: Appl Microbiol Biotechnol. 2024 Apr 24;108(1):306. doi: 10.1007/s00253-024-13136-z (PMC11043171; doi:10.1007/s00253-024-13136-z)
Supplement: Supplementary file 1 — Supplementary file1 (PDF 740 KB) [file 253_2024_13136_MOESM1_ESM.pdf]

Supplementary Material for Applied Microbiology and Biotechnology

An overview of the two-component system GarR/GarS role on antibiotic production in *Streptomyces coelicolor*

Rodrigo Cruz-Bautista[1], Augusto Zelarayan-Agüero[1], Beatriz Ruiz-Villafán[1], Adelfo Escalante-Lozada[2], Romina Rodríguez-Sanoja[1] and Sergio Sánchez[1*].

[1]Instituto de Investigaciones Biomédicas, Departamento de Biología Molecular y Biotecnología, Universidad Nacional Autónoma de México, Ciudad Universitaria, CdMx, 04510. México.

[2]Instituto de Biotecnología, Departamento de Ingeniería Celular y Biocatálisis. Universidad Nacional Autónoma de México. Ave. Universidad 2001, Cuernavaca, 62210. México.

Table S1 Strains and vectors used in this work. Also, the strains and vectors constructed in this work are presented.

**Strain and vector**	**Main characteristics and purpose**	**Reference**
E. coli DH5α	General cloning procedures. *glnV44 Φ80' lacZ(del)M15 recA1 relA1 endA1 hsdR17*	(Kieser et al. 2000)
E. coli BW25113	λ RED recombination strain.	(Gust et al. 2003)

	(Δ(*araD-araB*)*567*, Δ*lacZ4787*(::*rrnB-4*), *lacIp-4000*(lacIQ), λ-, *rpoS369*(Am), *rph-1*, Δ(*rhaD-rhaB*)568, *hsdR514*.<br>Containing plasmid pIJ790 *[oriR101], [repA101*(ts)*], araBp-gam-be-exo*.	
E. coli ET12567	Methylation deficient strain containing the non-transmissible plasmid pUZ8002.<br>Resistant to chloramphenicol and tetracycline.	(Gust et al. 2003)
E. coli JM110	Lacks both dam and dcm activity.<br>*rpsL* (*Str^r*) *thr leu thi-1 lacY galK galT ara tonA tsx dam dcm supE44 Δ*(*lac-proAB*) *[F traD36 proAB lacI^qZΔM15]*	(Yanisch-Perron et al. 1985)
S. coelicolor M145	Streptomycete laboratory model.	(Bentley et al. 2002)
Δ*garR*	*S. coelicolor* M145 mutant strain, lacking the two-component system response regulator *sco6162*.	This work.
Δ*garS*	*S. coelicolor* M145 mutant strain, lacking the two-component system sensor histidine kinase *sco6163*.	This work.
Δ*garR*/Δ*garS*	*S. coelicolor* M145 strain, lacking the complete two-component system *sco6162*/*sco6163*.	This work.
Δ*garR*-C	Mutant strain complemented with the vector containing the sequence of a 400 pb upstream *sco6163* and 3xFLAG in fusion with *sco6162* with pKU1021 as backbone (pKU-*P3X-6162*).	This work.
Δ*garS*-C	Mutant strain complemented with the vector containing the sequence of a 400 pb upstream *sco6163* and 3xFLAG in fusion with *sco613* with	This work.

	pKU1021 as backbone (pKU-*P3X-6163*).	
St1A9 cosmid	Supercos-1 cosmid containing the chromosomic DNA region from 6738815 to 6780399 of *S. coelicolor* A3(2).	(Redenbach, et al. 1996)
pKU1021	Integrative vectors carrying *attP*/*int* of bacteriophage ϕC31and the constitutively expressed promoter (rpsJp). Resistance genes *aac(3)I* and *aphII* (apramycin and kanamycin respectively).	(Komatsu et al. 2013)
pKU-*P3X-garR*	pKU1021 backbone without the *aac(3)I* gene and the *rpsJp* promoter. Contains 400 pb upstream *sco6163* and the 3xFLAG sequence in fusion with the *sco6162* gene.	This work.
pKU-*P3X-garS*	pKU1021 backbone without the *aac(3)I* gene and the *rpsJp* promoter. Contains 400 pb upstream *sco6163* and the 3xFLAG sequence in fusion with the *sco6163* gene.	This work.
pGEM-*garR*	Commercial (Promega) linearized vector for cloning PCR products containing the *sco6162* sequence of *S. coelicolor*.	This work.

Table S2 Primers used and designed in this work.

**Primer**	**Sequence**	**Purpose**	**Source**
fwd*garR*Targ	GCTCACCGCACCCCTTCCCAGGAAGTAGCACCGACCATGTGTAGGCTGGAGCTGCTTC	PCR targeting for *garR* deletion.	This work.
rv*garR*Targ	GTGGGCGGCCAGAGCCACGGGCGGTTCGGGGGACCGTCAATTCCGGGGATCCGTCGACC		
fwd*garS*Targ	GTGTCCCTGTTCTGGCGGATCTTCGGGCTCAACGCGGTGTGTAGGCTGGAGCTGCTTC	PCR targeting for *garS* deletion.	This work.
rv*garS*Targ	ACGGCTCGGGCAGGGACGGGTCGGACATGGTCGGTGCTAATTCCGGGGATCCGTCGACC		
fwd*garR*shIn	GTCCAGACCTGACCACGAGG	*garR* deletion verification.	This work.
rv*garR*shOut	CGAGCCGTCGTCGCATGCAC		
fwd*garS*shIn	GACGAGCCGTTCGATCCTGC	*garS* deletion verification.	This work.
fwd*garS*shOut	GGCCGCAGCTCCCACCGAGG		
XbaI*garR*_F	TGCTCTAGAATGTCCGACCCGTCCCTGCCCGAG	For cloning *garR* in pKU1021.	This work.

HindIII*garR*_R	CCCAAGCTTGGGGGACCGTCAGGGC TCGATGAG		
XbaI*garS*_F	TGCTCTAGAGTGGTGCTGGGCTTCG CCACG	For cloning *garS* in pKU1021.	This work.
HindIII*garS*_R	CCCAAGCTTTACTTCCTGGGAAGGG GTGCGGTG		
3xFLAG_F	GAATCCCATATGGACTACAAGGACC ACGACGGCGACTACAAGGACC ACGACA	For cloning *3xFLAG* in pKU-*garR* and pKU-*garS*.	This work.
3xFLAG_R	TGCTCTAGACTTGTCGTCGTCGTCCT TGTAGTCGATGTCGTGGTCCTT GTAGTCGC		
pKUPrpsJ_F	CCAGAGCGTTTTATCGAGACGAA	For sequencing pKU-3X-*garR* and *garS* constructions.	This work.
pKUHindIII_R	CGGATAACAATTTCACACAGGAAAC A		
F_Prom	ATCATGATATCGCGGGAGGCGTCGA GCGACAC	Amplification of 400 pb upstream *garS*.	This work.
R_Prom	CAAACATATGCGCGTTGAGCCCGAA GATCCGCC		
F_transcript	CATCGAGGCGGCGCACGAG	Transcript detection.	This work.

R_transcript	AAGATGCGGATCTTCGAGGGG		

Table S3 Orthologues used in the generation of predicted motifs for the TCS *sco6162/sco6163*.

	**KEGG Orthology database**	
	**Species**	**Entry**
1	*Streptomyces coelicolor*	SCO6163
2	*Streptomyces lividans*	SLIV_07320
3	*Streptomyces ambofaciens*	SAM23877_5894
4	*Streptomyces parvulus*	Spa2297_26125
5	*Streptomyces pactum*	B1H29_07570
6	*Streptomyces chartreusi*	CP983_39470
7	*Streptomyces glaucescens*	SGLAU_30990
8	*Streptomyces autolyticus*	BV401_15545
9	*Streptomyces cyaneogriseus*	TU94_26450
10	*Streptomyces leeuwenhoekii*	sle_15120
11	*Streptomyces rapamycinicus*	M271_02725
12	*Streptomyces malaysiensis*	SMALA_2434
13	*Streptomyces solisilvae*	I1A49_15245
14	*Streptomyces hygroscopicus* subsp. *jinggangensis* TL01	SHJGH_8288
15	*Streptomyces caniscabiei*	IHE65_08285
16	*Streptomyces tirandamycinicus*	DDW44_12625

17	*Streptomyces scabiei*	SCAB_6071
18	*Streptomyces deccanensis*	L3078_37260
19	*Streptomyces griseoviridis*	ELQ87_38770
20	*Streptomyces coeruleorubidus*	CP976_02145
21	*Streptomyces aurantiacus*	GCM10017557_03210
22	*Streptomyces galilaeus*	CP966_27755
23	*Streptomyces fungicidicus*	CNQ36_03645
24	*Streptomyces viridosporus*	CP969_32760
25	*Streptomyces cyanogenus*	S1361_34925
26	*Streptomyces cadmiisoli*	DN051_39135

Table S4. Primers used for qPCR experiments.

**Primer**	**Sequence**	**Product size (bp)**	**Source**
qactIIORF4_F	AAAGGAATATCGCGCACCTGGAAG	102	(Honma et al. 2021)
qactII-ORF4_R	GTTCCGGAATCATCGGCCCTATTC		
qredD_F	GACCTGGTGGACGAACTGTG	99	This work.
qredD_R	ACGCTCGTTGAGCACTTTC		
qredZ_F	TCTTCACCATGGACAGCATCGAC	191	(Honma et al. 2021)
qredZ_R	GGCATTGACTTCGGTGATGGTTC		

qhrdB_F	GCATGCTCTTCCTGGACCTCAT	93	(Romero-Rodríguez et al. 2016b)
qhrdB_R	TGGAGAACTTGTAGCCCTTGGTGTA		
qgarR_F	TCCTGATGCTGACGATGCAC	85	This work.
qgarR_R	ACCGACTTGAGCACGTATCC		

Table S5. Score for every interaction source and the combined score for the GarR interactome made with STRING.

Node 1	Node 2	Neighborhood on chromosome	Gene fusion	Phylogenetic cooccurrence	Homology	Coexpression	Experimentally Determined interaction	Database annotated	Automated textmining	Combined score
SCO5454	SCO6162	0.044	0.002	0.681	0	0.136	0.134	0	0	0.741
SCO5784	SCO6162	0.129	0	0.772	0	0.136	0.134	0	0.042	0.831
SCO5784	SCO6424	0	0	0.069	0.961	0	0	0.9	0	0.902
SCO5784	SCO6163	0	0	0.08	0.946	0	0	0.9	0	0.904
SCO6162	SCO6167	0.294	0	0	0	0.056	0	0	0.646	0.743
SCO6162	SCO6164	0.44	0	0	0	0	0	0	0.645	0.792
SCO6162	SCO6424	0.129	0	0.773	0	0.136	0.134	0	0.042	0.832
SCO6162	SCO6163	0.793	0	0.772	0	0.136	0.134	0	0.646	0.985
SCO6163	SCO6167	0.293	0	0.164	0	0.055	0	0	0.646	0.775
SCO6163	SCO6164	0.394	0	0.17	0	0	0	0	0.642	0.804
SCO6163	SCO6424	0	0	0.082	0.944	0	0	0.9	0	0.904
SCO6164	SCO6167	0.722	0	0	0	0	0	0	0.642	0.896

Table S6. The score for every interaction source and the combined score for the GarS interactome made with STRING.

Node 1	Node 2	Neighborhood on chromosome	Gene fusion	Phylogenetic cooccurrence	Homology	Coexpression	Experimentally determined interaction	Database annotated	Automated textmining	Combined score
SCO1370	SCO6163	0.059	0	0.637	0	0.136	0.134	0	0.042	0.71
SCO2216	SCO6163	0.059	0	0.63	0	0.136	0.134	0	0.042	0.704
SCO5455	SCO5784	0.059	0	0.63	0	0.136	0.134	0	0.042	0.704
SCO5455	SCO6163	0.059	0	0.654	0	0.136	0.134	0	0.042	0.724
SCO5784	SCO5881	0	0	0.629	0	0.136	0.134	0	0.17	0.738
SCO5784	SCO6162	0.129	0	0.772	0	0.136	0.134	0	0.042	0.831
SCO5784	SCO6424	0	0	0.069	0.961	0	0	0.9	0	0.902
SCO5784	SCO6163	0	0	0.08	0.946	0	0	0.9	0	0.904
SCO5784	SCO5785	0.793	0	0.773	0	0.136	0.134	0	0.042	0.96
SCO5785	SCO6163	0.129	0	0.773	0	0.136	0.134	0	0.042	0.832
SCO5785	SCO6424	0.129	0	0.773	0	0.136	0.134	0	0.042	0.832
SCO5881	SCO6163	0	0	0.653	0	0.136	0.134	0	0	0.717
SCO6162	SCO6167	0.294	0	0	0	0.056	0	0	0.646	0.743
SCO6162	SCO6164	0.44	0	0	0	0	0	0	0.645	0.792
SCO6162	SCO6424	0.129	0	0.773	0	0.136	0.134	0	0.042	0.832
SCO6162	SCO6163	0.793	0	0.772	0	0.136	0.134	0	0.646	0.985
SCO6163	SCO6167	0.293	0	0.164	0	0.055	0	0	0.646	0.775
SCO6163	SCO6164	0.394	0	0.17	0	0	0	0	0.642	0.804
SCO6163	SCO6424	0	0	0.082	0.944	0	0	0.9	0	0.904
SCO6164	SCO6167	0.722	0	0	0	0	0	0	0.642	0.896

Table S7 Predicted genes to be regulated by the TCS GarR/GarS for each predicted motif.

	**Motif 1**			
	**SCO #**	**Description**	**Function**	**E-value**
1	SCO6164	Hypothetical protein SC1A9.28c	Translation	2.6E-12
2	SCO6163	Putative sensor kinase	Signal transduction mechanisms	2.6E-12
3	SCO5783	Conserved hypothetical protein SC4H2.04c	Inorganic ion transport and metabolism	5.1E-09
4	SCO6489	Conserved hypothetical protein	Mobilome: prophages	0.085
5	SCO3406	Conserved hypothetical protein	Mobilome: prophages	0.16
6	SCO6425	Hypothetical protein	General function prediction only	0.5
7	SCO6998	Putative cytochrome P450.	Secondary metabolites biosynthesis	0.67
8	SCO5986	Putative oxidoreductase	Energy production and conversion	1.4
9	SCO3409	Putative inorganic pyrophosphatase	Energy production and conversion	1.6
10	SCO3408	Conserved hypothetical protein	Mobilome: prophages	1.6
11	SCO7057	Putative esterase	Defense mechanisms	1.6
12	SCO5313	Possible membrane protein	Function unknown	2.1
13	SCO5428	Putative integral membrane transport protein	Carbohydrate transport and metabolism	3.2
14	SCO0923	Putative reductase flavoprotein subunit	Energy production and conversion	3.4

15	SCO0292	Putative sugar ABC-transporter integral membrane protein	Carbohydrate transport and metabolism	3.7
16	SCO7121	Putative secreted protein	Inorganic ion transport and metabolism	4.5
17	SCO7190	Hypothetical protein	General function prediction only	4.5
18	SCO0533	Putative sugar transporter membrane protein	Carbohydrate transport and metabolism	4.8
19	SCO2842	Putative membrane protein.	Carbohydrate transport and metabolism	4.9
20	SCO5651	Hypothetical protein SC6A9.16	General function prediction only	4.9
21	SCO1353	Putative transcriptional regulator	Regulatory protein	5.9
22	SCO7508	Conserved hypothetical protein	Mobilome: prophages	5.9
23	SCO7688	Conserved hypothetical protein	Mobilome: prophages	6
24	SCO5212	3-Phosphoshikimate 1-carboxyvinyltransferase	Amino acid transport and metabolism	6.7
25	SCO2783	Putative monooxygenase	Energy production and conversion	7.1
26	SCO0558	Hypothetical protein SCF73.05c	Carbohydrate transport and metabolism	7.1
27	SCO3919	Putative lysR-family transcriptional regulator	Regulatory protein	8
28	SCO0132	Probable transcriptional regulator	Regulatory protein	8.9
29	SCO0131	Putative secreted protein	Inorganic ion transport and metabolism	8.9
30	SCO3587	Putative regulatory protein	Regulatory protein	8.9

31	SCO3612	Putative membrane protein	Transcription	9.4
	**Motif 2**			
	**SCO#**	**Description**	**Function**	**E-value**
1	SCO6164	Hypothetical protein SC1A9.28c	Translation	3.7E-11
2	SCO6163	Putative sensor kinase	Signal transduction mechanisms	3.7E-11
3	SCO6708	Putative membrane protein	Transcription	0.055
4	SCO6707	Putative DNA ligase	Replication	0.055
5	SCO0416	Probable integral membrane protein	Function unknown	0.78
6	SCO3886	Putative partitioning or sporulation protein	Cell motility	1.6
7	SCO1771	Hypothetical protein	General function prediction only	2
8	SCO1186	Putative lacI-family transcriptional regulator	Regulatory protein	2.6
9	SCO2815	Putative TetR-family transcriptional regulator	Regulatory protein	2.6
10	SCO2814	Putative methyltransferase	Coenzyme transport and metabolism	2.6
11	SCO2441	Hypothetical protein SCC24.12	Function unknown	2.8
12	SCO2214	Putative reductase	General function prediction only	3.1
13	SCO0818	Putative ABC transporter ATP-binding protein	General function prediction only	4.1
14	SCO5168	Putative integral membrane protein	Posttranslational modification	4.1
15	SCO6563	Putative integral membrane transporter	Inorganic ion transport and metabolism	5.2
16	SCO6861	Protein kinase-like protein.	Signal transduction mechanisms	5.6
17	SCO2228	Alpha-glucosidase	Carbohydrate transport and metabolism	6.2

18	SCO7260	Possible membrane protein	Function unknown	6.5
19	SCO3915	Putative transmembrane efflux protein	Carbohydrate transport and metabolism	6.9
20	SCO6712	Putative copper oxidase	Inorganic ion transport and metabolism	7.6
21	SCO6711	Conserved hypothetical protein	Mobilome: prophages	7.6
22	SCO1370	Putative two component system DNA binding response regulator	Transcription	8.1
23	SCO4119	Putative NADH dehydrogenase	Energy production and conversion	8.1
24	SCO4118	putative tetR-family transcriptional regulator	Regulatory protein	8.1
25	SCO2297	Hypothetical protein SCC30.05	Inorganic ion transport and metabolism	8.5
26	SCO3332	Putative membrane protein	Transcription	9.1
27	SCO0551	Putative histidine kinase protein	Signal transduction mechanisms	9.3
28	SCO1878	Putative secreted protein	Inorganic ion transport and metabolism	9.3
29	SCO7794	Putative TetR-family transcriptional regulator	Regulatory protein	9.8
30	SCO7792	Hypothetical protein	General function prediction only	9.8
31	SCO7793	Hypothetical protein	General function prediction only	9.8
32	SCO1427	Hypothetical protein SC6D7.12c	Energy production and conversion	10
	**Motif 3**			
	**SCO#**	**Description**	**Function**	**E-value**

1	SCO6164	Hypothetical protein SC1A9.28c	Translation	2.2E-09
2	SCO6163	putative sensor kinase	Signal transduction mechanisms	2.2E-09
3	SCO5520	Delta-1-pyrroline-5-carboxylate dehydrogenase	Energy production and conversion	0.0086
4	SCO6425	Hypothetical protein	General function prediction only	0.089
5	SCO6424	Putative two-component system sensor protein	Signal transduction mechanisms	0.089
6	SCO4579	Putative integral membrane protein	Posttranslational modification	0.92
7	SCO3537	Putative DNA-binding protein	Transcription	1.3
8	SCO0502	Putative membrane protein	Transcription	1.3
9	SCO0501	Putative secreted protein	Inorganic ion transport and metabolism	1.3
10	SCO2918	Putative nicotinamidase	Coenzyme transport and metabolism	3.6
11	SCO0722	Putative hydrolase	Energy production and conversion	3.6
12	SCO1867	Putative hydroxylase	General function prediction only	3.7
13	SCO7404	Conserved hypothetical protein SC10G8.32c.	Energy production and conversion	4.4
14	SCO1807	Putative integral membrane protein	Posttranslational modification	5.6
15	SCO1806	Putative ABC transporter ATP-binding protein	General function prediction only	5.6
16	SCO4094	Conserved hypothetical protein SCD25.30	Posttranslational modification	5.9
17	SCO4093	Putative integral membrane protein	Posttranslational modification	5.9
18	SCO5784	Putative two-component sensor	Signal transduction mechanisms	7.4

19	SCO5783	Conserved hypothetical protein SC4H2.04c	Inorganic ion transport and metabolism	7.4
20	SCO7342	Hypothetical protein SC4G10.21.	Carbohydrate transport and metabolism	7.6
21	SCO7341	Putative RNA polymerase secondary sigma factor	Transcription	7.6

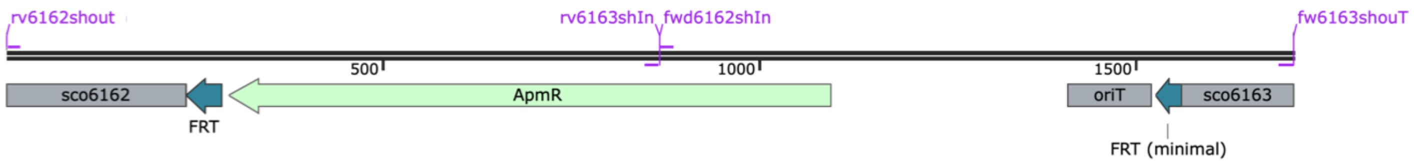

Fig. S1 Map of the *sco6163* gene disruption with the apramycin resistance gene in the mutant strains. The primer sets used to corroborate the deletion by PCR are marked.

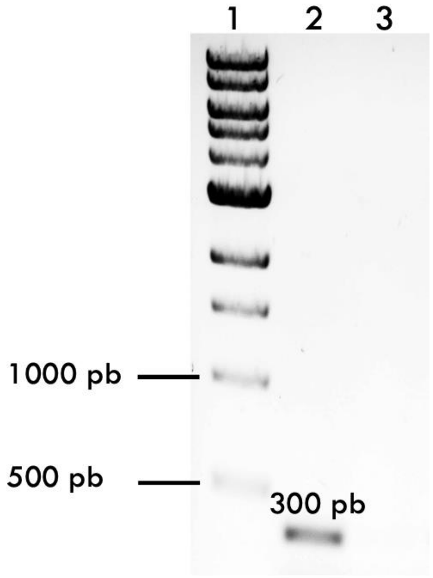

Fig. S2 The agarose gel (0.8%) electrophoresis was run in TAE 1X, for 1 h, at 90V. Lane 1: 1 kb DNA Ladder NEB® Lane 2: Product of the forward (F) and reverse (R) transcript primers which amplify the intergenic region of 8 bp between the *garR*/*garS* genes. Lane 3: Negative control with F and R transcript primers with a RNA sample without RT-PCR.

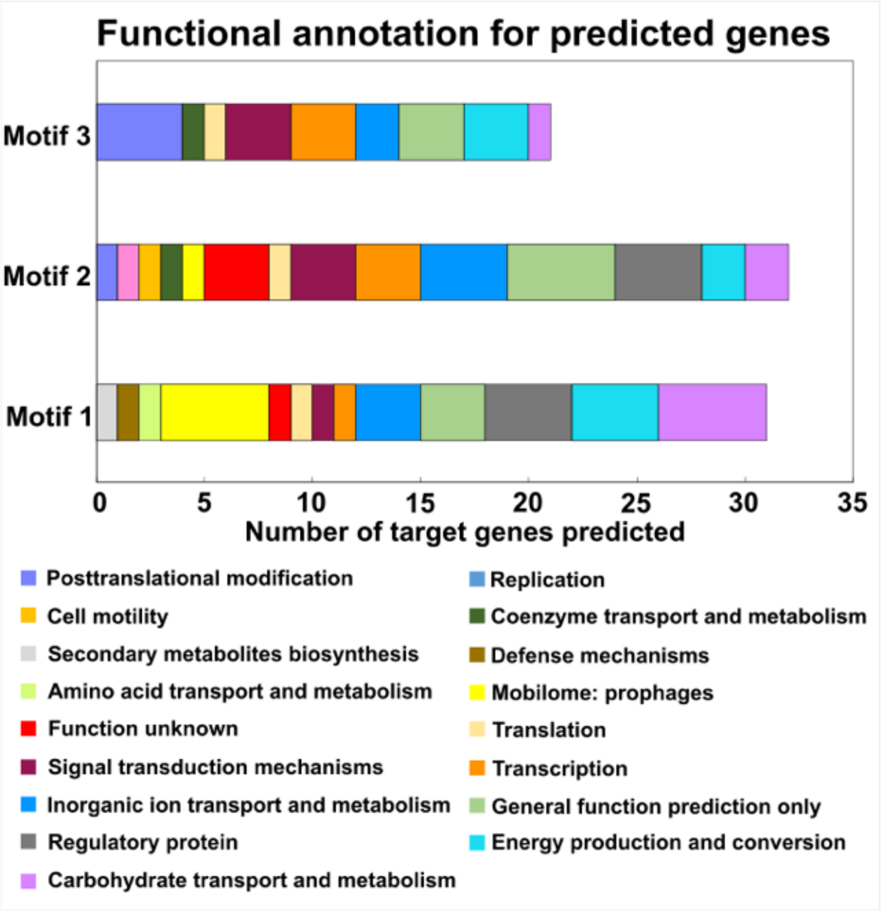

Fig. S3 Functional annotation of the predicted genes according to the Clusters of Orthologous Groups of proteins (COGs) database. The number of the classified genes for each motif (horizontal axis) are shown in different colors and the respective functions are listed below the graphic according to the color code.

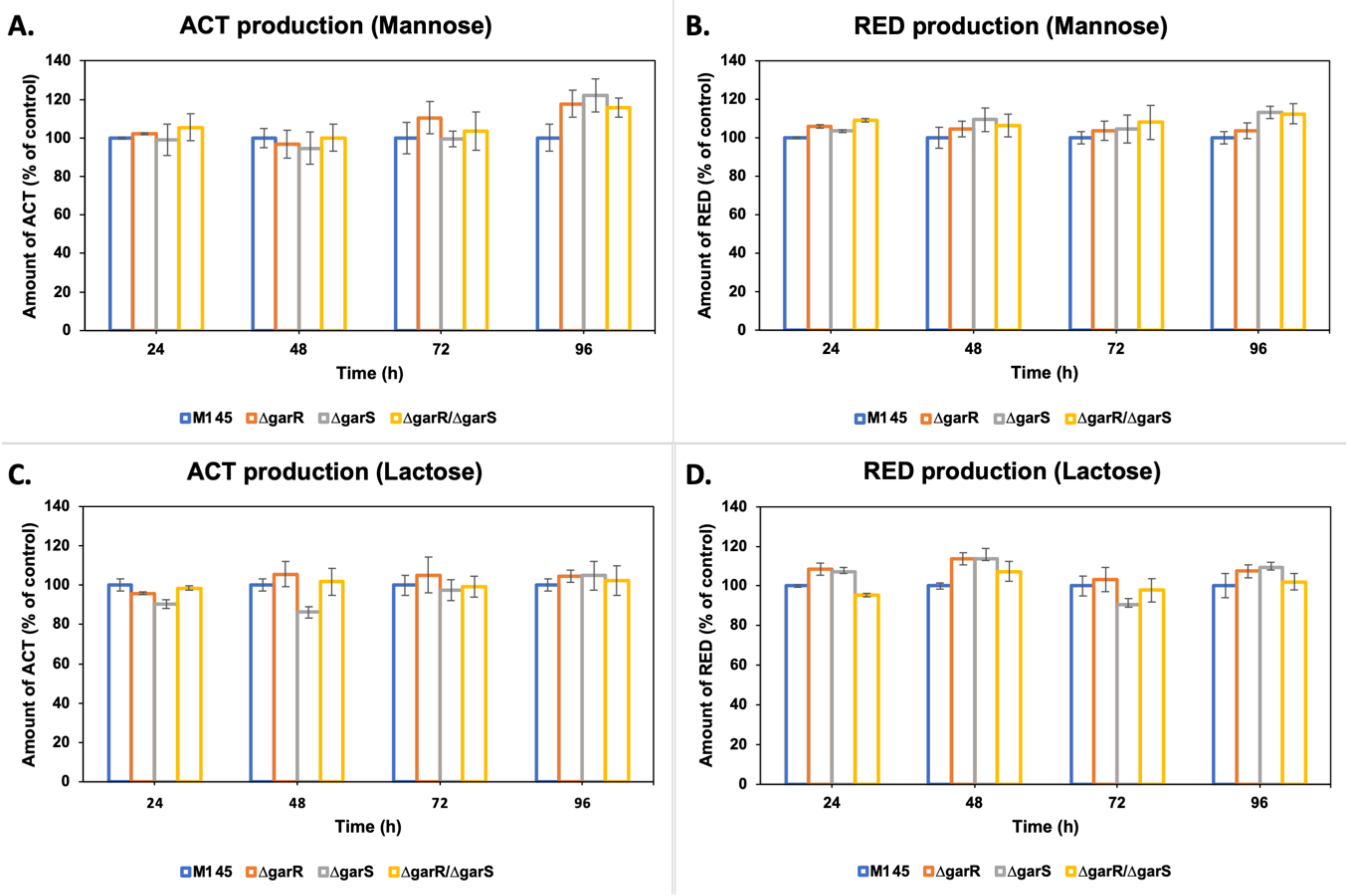

Fig. S4 Percentage of produced ACT and RED by the M145 (blue lines), Δ*garR* (orange lines), Δ*garS* (grey lines) and Δ*garR*/Δ*garS* (yellow lines) strains grown in NMMP supplemented with mannose (**A** and **B**) or lactose (**C** and **D**) (0.5% each) as the sole carbon source and harvested at 24, 48, 72 and 96 h. The graphic illustrates the percentage of the amount of antibiotic produced by each strain relative to the M145 control.

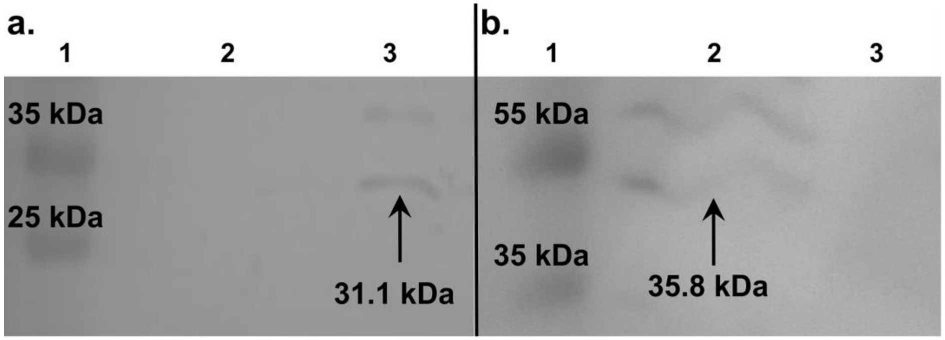

Fig. S5 Western-blot analysis of the 3xFLAG tagged proteins. (**a**)**:** GarR (lane 1: Prestained protein PageRulerTM (26616), lane 2: negative control and lane 3: 3xFLAG-GarR). (**b**)**:** GarS (lane 1: Prestained protein PageRulerTM (26619) lane 2: negative control and lane 3: 3xFLAG-GarS). The arrows indicate the expected proteins and their weight (kDa), as referred to the protein ladder.
